# Comparative analysis of the human saliva microbiome from different climate zones: Alaska, Germany, and Africa

**DOI:** 10.1186/s12866-014-0316-1

**Published:** 2014-12-17

**Authors:** Jing Li, Dominique Quinque, Hans-Peter Horz, Mingkun Li, Margarita Rzhetskaya, Jennifer A Raff, M Geoffrey Hayes, Mark Stoneking

**Affiliations:** Max Planck Institute for Evolutionary Anthropology, Deutscher Platz 6, D-04103 Leipzig, Germany; Max Planck Independent Research Group on Population Genomics, Chinese Academy of Sciences and Max Planck Society (CAS-MPG) Partner Institute for Computational Biology, Shanghai Institutes for Biological Sciences, Chinese Academy of Sciences, 320 Yueyang Road, Shanghai, 200031 China; Division of Virology, Institute of Medical Microbiology, RWTH Aachen University Hospital, Pauwelsstrasse 30, D-52057 Aachen, Germany; Division of Endocrinology, Metabolism, and Molecular Medicine, Department of Medicine, Northwestern University Feinberg School of Medicine, Chicago, IL 60611 USA; Department of Anthropology, Northwestern University, Evanston, IL 60208 USA; Center for Genetic Medicine, Northwestern University Feinberg School of Medicine, Chicago, IL 60611 USA; Current address: Department of Genetics, Harvard Medical School, 77 Louis Pasteur Avenue, Boston, MA 02115 USA; Current address: Department of Anthropology, University of Texas, Austin, TX 78712 USA

**Keywords:** Saliva, Microbial community, Humans

## Abstract

**Background:**

Although the importance of the human oral microbiome for health and disease is increasingly recognized, variation in the composition of the oral microbiome across different climates and geographic regions is largely unexplored.

**Results:**

Here we analyze the saliva microbiome from native Alaskans (76 individuals from 4 populations), Germans (10 individuals from 1 population), and Africans (66 individuals from 3 populations) based on next-generation sequencing of partial 16S rRNA gene sequences. After quality filtering, a total of 67,916 analyzed sequences resulted in 5,592 OTUs (defined at ≥97% identity) and 123 genera. The three human groups differed significantly by the degree of diversity between and within individuals (e.g. beta diversity: Africans > Alaskans > Germans; alpha diversity: Germans > Alaskans > Africans). UniFrac, network, ANOSIM, and correlation analyses all indicated more similarities in the saliva microbiome of native Alaskans and Germans than between either group and Africans. The native Alaskans and Germans also had the highest number of shared bacterial interactions. At the level of shared OTUs, only limited support for a core microbiome shared across all three continental regions was provided, although partial correlation analysis did highlight interactions involving several pairs of genera as conserved across all human groups. Subsampling strategies for compensating for the unequal number of individuals per group or unequal sequence reads confirmed the above observations.

**Conclusion:**

Overall, this study illustrates the distinctiveness of the saliva microbiome of human groups living under very different climatic conditions.

**Electronic supplementary material:**

The online version of this article (doi:10.1186/s12866-014-0316-1) contains supplementary material, which is available to authorized users.

## Background

The human body hosts about 100 trillion bacterial cells, around 10 times more than the number of human cells. Bacteria have been shown to play an important role in human health, affecting weight, immune response, nutrient absorption, and other aspects [1,2]. More than 1000 bacterial species have been found in and on the human body, some of which promote health while others contribute to illness [3,4]. However, recent studies have shown that instead of distinct microbial species being either beneficial or harmful for human health, perturbations in the global balance of the microbiome might play a more important role [5,6]. Therefore, it is important to understand what constitutes a “normal” bacterial community in the healthy human body [7-9], and how this might vary across populations.

The oral cavity is a major gateway to the human body, and microorganisms colonizing the oral cavity have the possibility to spread to neighboring sites and influence the gastrointestinal microbiome[10]. In addition, while mostly harmless in their primary (oral) habitat, oral bacteria have been linked with systemic, life-threatening disorders, e.g. cardiovascular disease, stroke, preterm birth, and pneumonia [11-16]. Hence the oral microbiome significantly affects human health. Moreover, the oral ecological system is influenced by external factors, such as food, drink, living temperature and humidity, and oral hygiene measures [17-19] and previous studies have shown a high degree of inter-individual variation in the oral microbiome [20,21]. While the overall oral health/disease status is a principal determinant of the oral microbial community compositions [20,22], comparatively little is known about how the structure and diversity of the oral microbiome varies across healthy human hosts of different ethnic or environmental origin.

We previously analyzed the saliva microbiome, via sequencing of a part of the16S ribosomal RNA (rRNA) gene, from 120 healthy individuals (10 individuals from each of 12 worldwide locations) and found a significant association between variation in the saliva microbiome and the distance of each location from the equator [21]. Furthermore, we found that the saliva microbiome of Batwa Pygmies, a former hunter-gatherer group from Africa, is much more diverse than the saliva microbiome of two agricultural African groups, most likely because of their different lifestyle and diet [23]. Other studies have found signatures of ethnicity in the oral microbiome [24]. Hence, both environment and geography may play a role in defining a “healthy” human oral microbiome. Here, we present a comparative analysis of the salivary microbiome diversity of three different human groups living under different climatic conditions. We obtained new data from 76 native Alaskans from four different geographic locations, all sampled from the northern extremity of the North American continent, as well as from ten individuals from Germany. For comparison, we also analyzed previously published data from 66 individuals from three different African populations located near the equator [23]. In this study, we are able to compare the salivary microbiome composition of different human populations living under very different climatic conditions and geographic locations.

## Results

### Saliva microbiome diversity at the genus level

After removing sequence reads less than 200 bp, quality filtering and chimeric checking via the AmpliconNoise pipeline [25], a total of 20909 sequences from the native Alaskans (Additional file 1: Figure S1) and 4618 sequences from the Germans remained for analysis. For the native Alaskan group 96.2% of the sequences could be assigned to a specific genus, while 3.8% were assigned as unknown by the Classifier in the RDP website [26]; for the German group, 96.9% of the sequences were assigned to specific genera while 3.1% of the sequences were assigned as unknown (Table 1).

**Table 1:** **Statistics for the microbiome diversity in native Alaskans (Atqasuk, Barrow, Nuiqsut, and Wainwright), Germans, and Africans (BP [Batwa Pygmies from Uganda], DRC [Democratic Republic of the Congo], and SL [Sierra Leone])**

**Group**	**Number of Individuals**	**Number of Sequences**	**Number of OTUs**	**Unknown (%)**	**Number of Genera**	**Variance between individuals (%)**	**Variance within individuals (%)**
Atqasuk	14	2661	807	4.5	45	13.43	86.57
Barrow	40	10937	2015	3.6	62	10.21	89.79
Nuiqsut	13	2972	853	3.7	41	7.60	92.40
Wainwright	9	2905	862	4.0	47	6.75	93.25
Germans	10	4388	887	3.1	58	2.32	97.68
BP	38	22948	3115	11.0	100	13.81	86.19
DRC	15	4503	703	9.9	41	43.75	56.25
SL	13	16602	1888	11.2	57	35.17	64.83
Africans	66	44053	4145	11.0	108	25.97	74.03
Alaskans	76	19475	2886	3.8	73	9.69	90.31
Germans	10	4388	887	3.1	58	2.21	97.79
Total	152	67916	5592	8.4	123	22.03	77.97

The number of different bacterial genera detected in each native Alaskan group ranged from 41 to 62, compared to 58 genera in the Germans and 41 to 100 genera in the African groups. We next carried out an analysis of molecular variance (AMOVA) at the genus level in order to investigate how much of the total variation in the saliva microbiome is due to differences within vs. among individuals from each group. The results (Table 1) indicate that the Barrow, Nuiqsut and Wainwright groups have similar apportionments of variation (variance between individual: 6.75 - 10.21% and variance within individuals: 89.79 - 93.25%), while the group from Atqasuk has slightly more differentiation between individuals (13.43%) and correspondingly less variance within individuals (86.57%). Thus, the group from Atqasuk is characterized by a higher heterogeneity of the salivary microbiome among individuals, compared to the other three native Alaskan groups. By contrast, the German group was characterized by very little variance among individuals (only 2.32%), while the African groups were characterized by much more variance among individuals (13.81 – 43.75%). When introducing an additional hierarchical level into the AMOVA by grouping individuals from the same population, the variance component among the four native Alaskan groups (−0.17%) was not significantly different from zero, indicating that the saliva microbiomes of the native Alaskan groups have similar compositions. By contrast, a variance component of 4.06% was observed among the three African groups, which is significantly different from zero. Thus, the composition of the saliva microbiome does differ significantly among the African groups. Overall, when grouped together according to climatic region (Table 1), the German group showed the smallest variance among individuals (2.21%), followed by native Alaskans (9.69%), and then Africans with the highest variance among individuals (25.97%).

In an attempt to further elucidate differences in diversity at the genus level, we calculated the within individual diversity (also called alpha diversity) using the Shannon-Weaver index [27], and inter-individuals’ diversity (or beta diversity) using the Sørensen index [28] (Additional file 2: Figure S2). Germans showed the highest alpha diversity and the lowest beta diversity. The four Alaskan groups had intermediate values for both the alpha diversity and the beta diversity. By contrast, the two agricultural African groups (Democratic republic of Congo [DRC] and Sierra Leone [SL]) showed the lowest alpha diversity values but comparatively high beta diversity values, while the hunter-gatherer Batwa Pygmies from Uganda [BP] group showed a significantly higher alpha diversity and also significantly lower beta diversity. Overall, these results are consistent with the AMOVA results.

To further investigate similarities and differences in the saliva microbiome among the eight populations, we used the ANOSIM analysis, based on permutation tests of the Sørensen index matrix for all individuals. This analysis indicated no significant differences among the four native Alaskan groups (ANOSIM statistic: R = −0.0935, P value = 0.7386, 10000 permutations) or between native Alaskans and Germans (P value = 0.7324) but there were significant differences between native Alaskans and Africans (P value = 0.0001) as well as between Germans and Africans (P value = 0.0001). These results indicate that native Alaskans and Germans are more similar to each other than to Africans in their saliva microbiome composition at the genus level.

### Abundance distribution of the saliva microbiome

**Figure 1:**
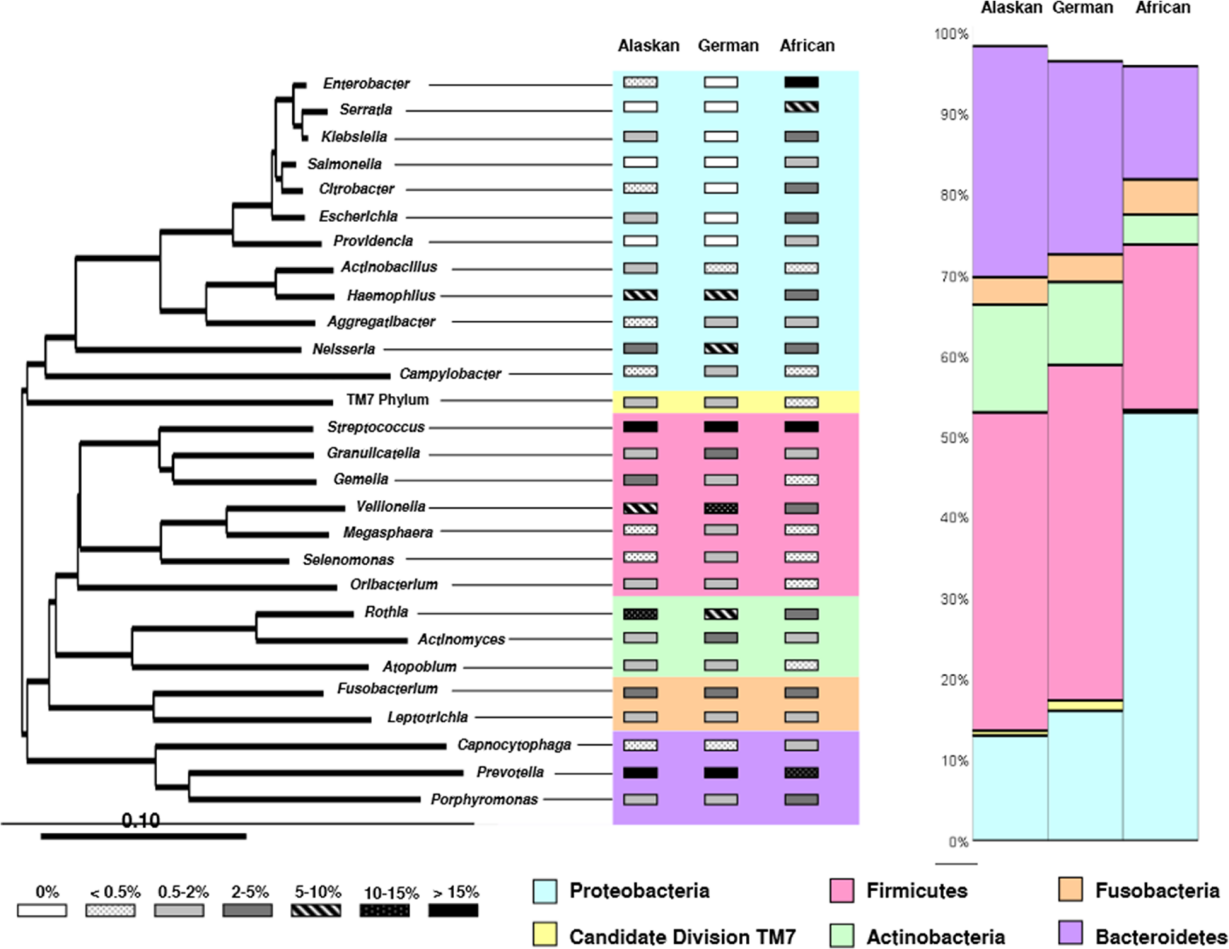
**Relative abundance of predominant genera (>0.5%) among native Alaskans, Germans and Africans.** The phylogenetic tree was calculated with representative full-length sequences, and the scale bar represents evolutionary distance (10 substitutions per 100 nucleotides). Bacterial phyla are indicated by different colors; the vertical bars on the right of each plot indicate the relative abundance of each phylum, as designated by the colors.

A heat plot of the abundance distribution of the genera (Additional file 3: Figure S3) shows that the native Alaskan group and the German group shared a higher number of genera than either group with the African group. The phylogenetic distribution of those genera with more than 0.5% abundance in at least one group are displayed in Figure 1, while the exact values and pairwise significance values are given in Additional file 4: Table S1. The most common phyla are *Actinobacteria* (A), *Bacteroidetes* (B), *Firmicutes* (F), *Fusobacteria* (Fu), *Proteobacteria* (P) and TM7 (T), with an abundance rank order F > B > P > A > Fu > T for native Alaskans and Germans, and P > F > B > Fu > A > T for Africans. At the genus level, there were 13 genera (covering all six phyla) out of the 28 common genera with comparable abundance in both native Alaskans and Germans. Conversely, native Alaskans and Africans shared only six genera (*Neisseria*, *Campylobacter, Granulicatella, Megasphaera, Selenomonas, Actinomyces*) with comparable abundance while Germans and Africans shared only three genera (*Actinobacillus*, *Aggregatibacter, Capnocytophaga,*), see Additional file 4: Table S1.Three genera (*Streptococcus, Fusobacterium, and Leptotrichia*) were of similar abundance in all three groups, and seven genera (*Enterobacter, Escherichia, Citrobacter, Gemella, Klebsiella, Rothia, and Veillonella*) were of different abundances in all three groups.

### Correlation analysis based on abundance distribution

To further investigate similarities and differences among the groups in the composition of the saliva microbiome, we calculated correlation coefficients for the distribution of genera detected between each pair of individuals, both within and between groups (Additional file 5: Figure S4). The average correlation coefficient among the four native Alaskan groups was 0.64, and none of the pairwise comparisons of the distribution of correlation coefficients between groups were significantly different (Mann–Whitney U tests, all p-values >0.05). The average correlation among Germans was 0.73, and between native Alaskans and Germans was 0.64, which was not significantly different (p = 0.2) from the average correlations within native Alaskans or Germans. However, the average correlation between native Alaskan groups and African groups was 0.49, and between Germans and the African groups was 0.49, both of which are significantly lower than the correlations within groups (p = 0.0007 and 0.0001, respectively).

### Phylogenetic analysis

**Figure 2:**
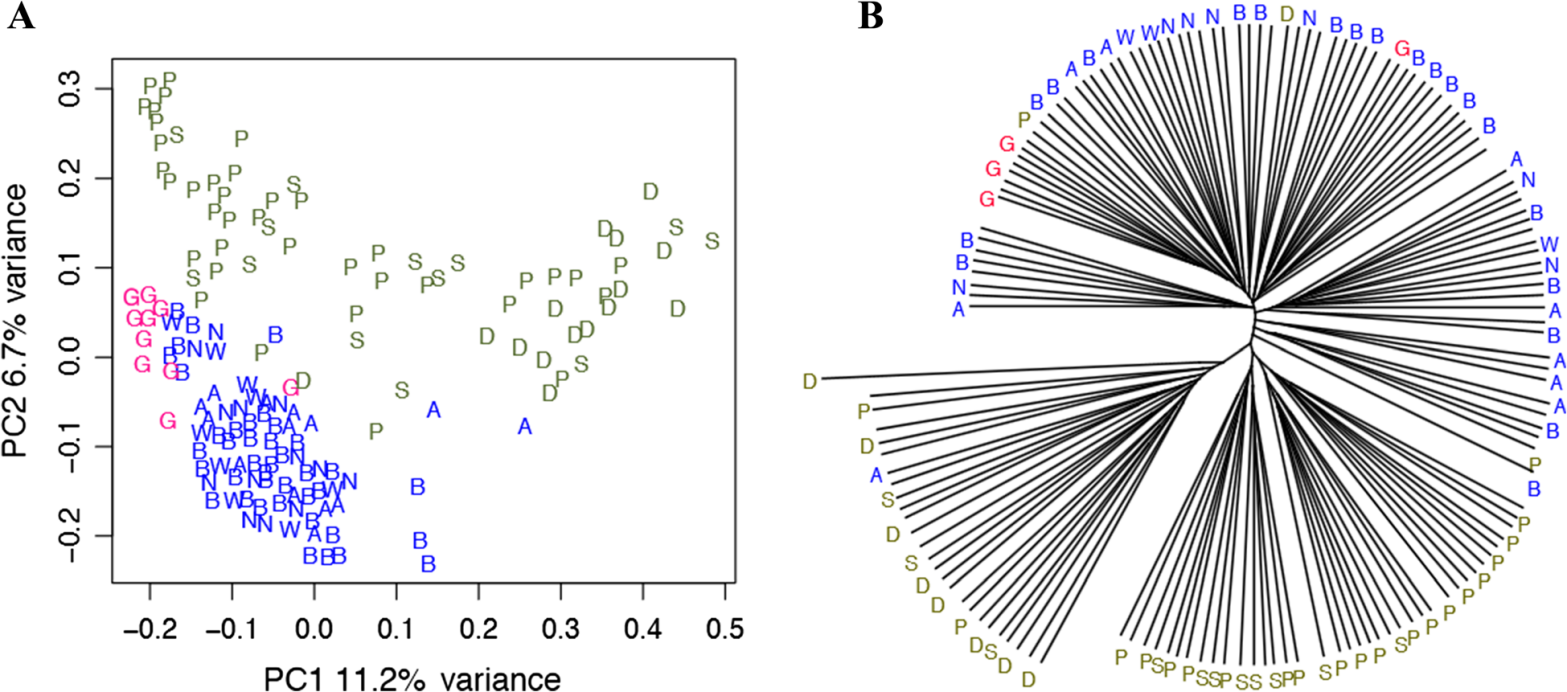
**Analyses based on the UniFrac distances: (A) principal coordinates analysis, and (B) UPGMA tree.** Each colored letter is an individual; colors represent regions (Blue, native Alaskans; red, Germans; green, Africans) and letters designate individual groups (A: Atqasuk; B: Barrow; N: Nuiqsut; W: Wainwright; G: Germans; D: DRC; S: SL; P: Batwa Pygmies).

We used the UniFrac metric to calculate a distance between each pair of individuals, based on their shared proportion of the sequence phylogeny. The result of principal coordinates analysis (PCoA) based on unweighted UniFrac distances is shown in Figure 2A. Although the two principal coordinates only account for 18% of the variance, the Africans are nonetheless largely (but not completely) separated from the native Alaskans and Germans, while the Germans are intermingled with the native Alaskans, with some tendency toward separation in PC2.

We also constructed an unweighted pair-group method with arithmetic means (UPGMA) tree based on the unweighted UniFrac distances (Figure 2B). With just a few exceptions, the Africans are clustered separately from the other groups, while the Germans cluster within the native Alaskans. Moreover, while there is some tendency for the Batwa Pygmies to cluster separately from the other African groups in both the PCoA and the UPGMA tree, the four native Alaskan groups do not show any differences from one another in either analysis. Overall, these results indicate that native Alaskans and Germans are quite similar to one another in saliva microbiome composition, but both differ significantly from the saliva microbiome of Africans.

### Bacterial interaction based on partial correlation analysis

**Figure 3:**
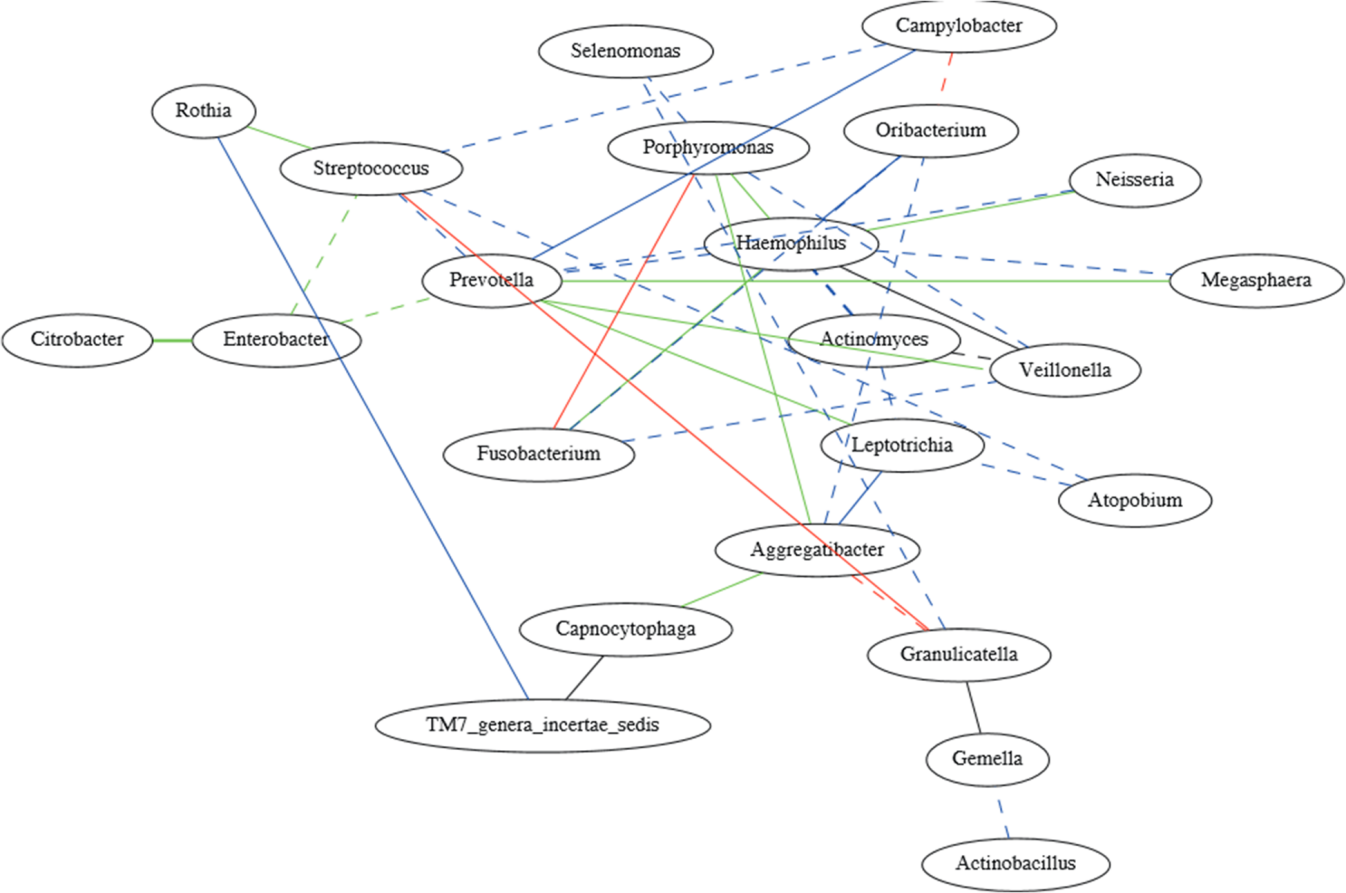
**Partial correlation network constructed by the frequency abundance of 28 common genera (frequency >0.5% in at least one regional groups) in 152 individuals from Alaska, Germany, and Africa.** In this figure, the solid lines represent positive correlations, and the dashed lines represent negative correlations. The line color indicates the shared correlation between different groups; green: correlation shared between Alaskans and Africans, blue:correlation shared between Alaskans and Germans, red: correlation shared between Germans and Africans, black: correlations shared among all three groups.

In addition to investigating the inter-subject and inter-group variability of oral microorganisms in human populations from very diverse geographical regions, we also investigated the possible existence of stable bacterial interactions, as these would additionally aid in understanding community structure and ecological interactions. We constructed partial correlation networks to visualize the direct or indirect relationships among bacteria for Alaskans, Germans, and Africans separately. Significant correlations were found for 147 pairs of bacterial genera of which the majority (i.e. 107) were uniquely present in only one human group: 48 in the Alaskan group, 37 in the German group, and 22 in the African group. Of the remaining 40 pairs (see Figure 3), 12 were shared between the Alaskan and the African group, 20 between the Alaskan and German group, and 4 between the German and African group. Four pairs of bacterial genera were shared between all three human populations, namely *Actinomyces-Veillonella, Gemella-Granulicatella*, *Haemophilus-Veillonella*, and *Capnocytophaga-TM7_genera_incertae_sedis*. Despite the high number of unique interactions at the genus level, the assignment of correlating genera to the phylum level showed a fairly consistent proportion of either phylum in the case of positive interactions (Additional file 6: Table S2). However, the number of negative interactions was much lower in the African group compared to the Alaskan and German group (Additional file 6: Table S2, Additional file 7: Figure S5).

### Saliva microbiome diversity at OTU level

**Figure 4:**
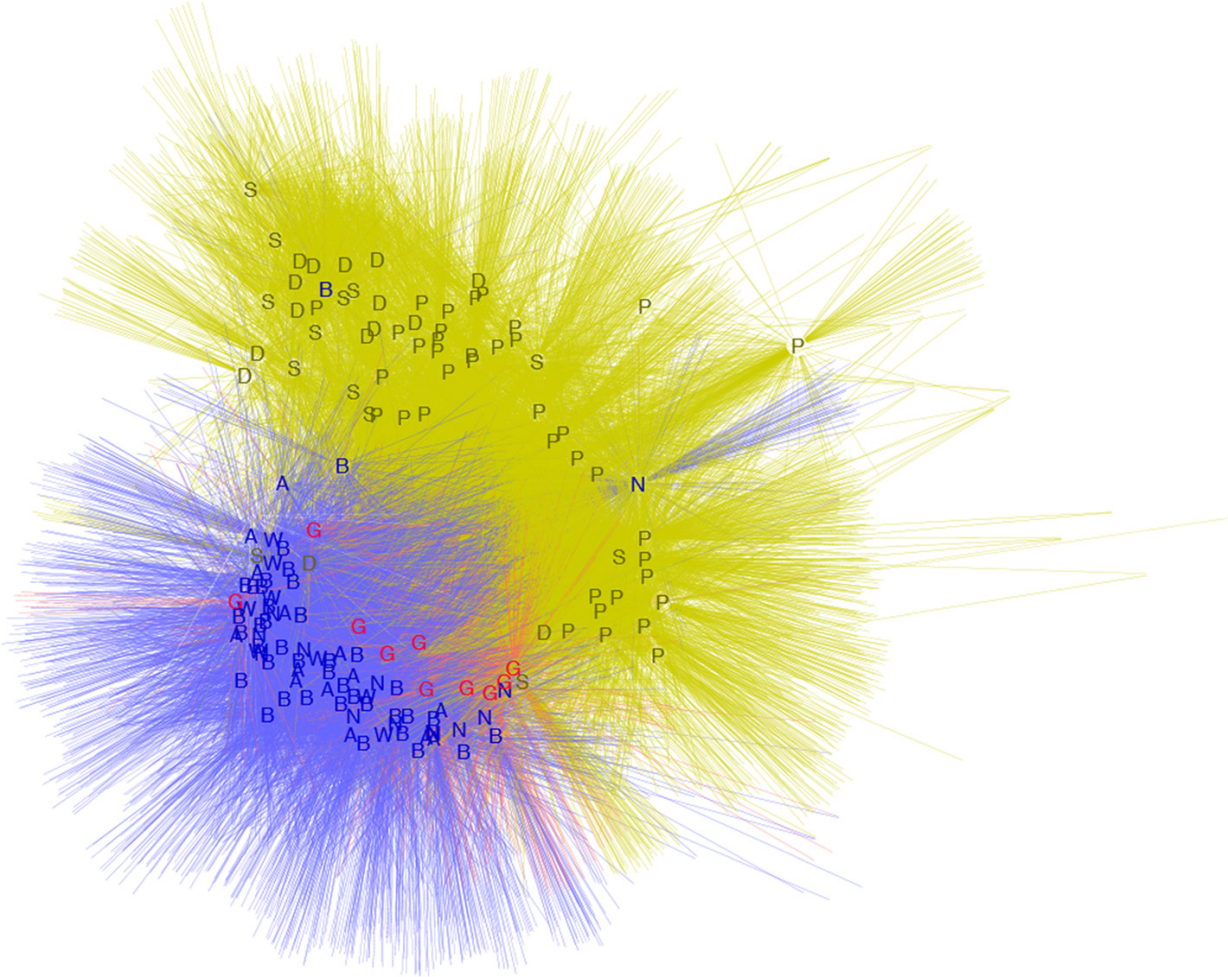
**Network relating individuals and OTUs.** Each colored letter is an individual; colors represent regions (Blue, native Alaskans; red, Germans; green, Africans) and letters designate individual groups (A: Atqasuk; B: Barrow; N: Nuiqsut; W: Wainwright; G: Germans; D: DRC; S: SL; P: Batwa Pygmies).

We also compared the salivary microbiome diversity among the different groups at the OTU (operational taxonomic unit) level, with OTUs defined by collapsing all sequences that were more than 97% identical to account for potential sequencing errors. Network analysis based on OTUs supported a clear clustering of the native Alaskan group distinct from the African group, with the German group in between but closer to the native Alaskans (Figure 4). Hence, as seen previously with analyses at the genus level, the OTU network confirmed a closer relationship between the salivary microbiomes of native Alaskans and Germans compared to the Africans.

To further compare the similarity of the salivary microbiome at the OTU level, we calculated the correlation coefficient based on OTU abundance among all individuals (Additional file 8: Figure S6). The average correlation coefficient within the four native Alaskan groups is 0.17. Conversely, the average correlation coefficient within three African groups is 0.09, and that within the German group is 0.33. The correlation coefficient between native Alaskans and the German group is 0.18, that between Alaskans and Africans is 0.07, and that between Germans and Africans is 0.10. Consistent with the previous analyses, the correlations at the OTU level further document more similarity between the salivary microbiomes of native Alaskans and Germans than between either group and Africans.

**Figure 5:**
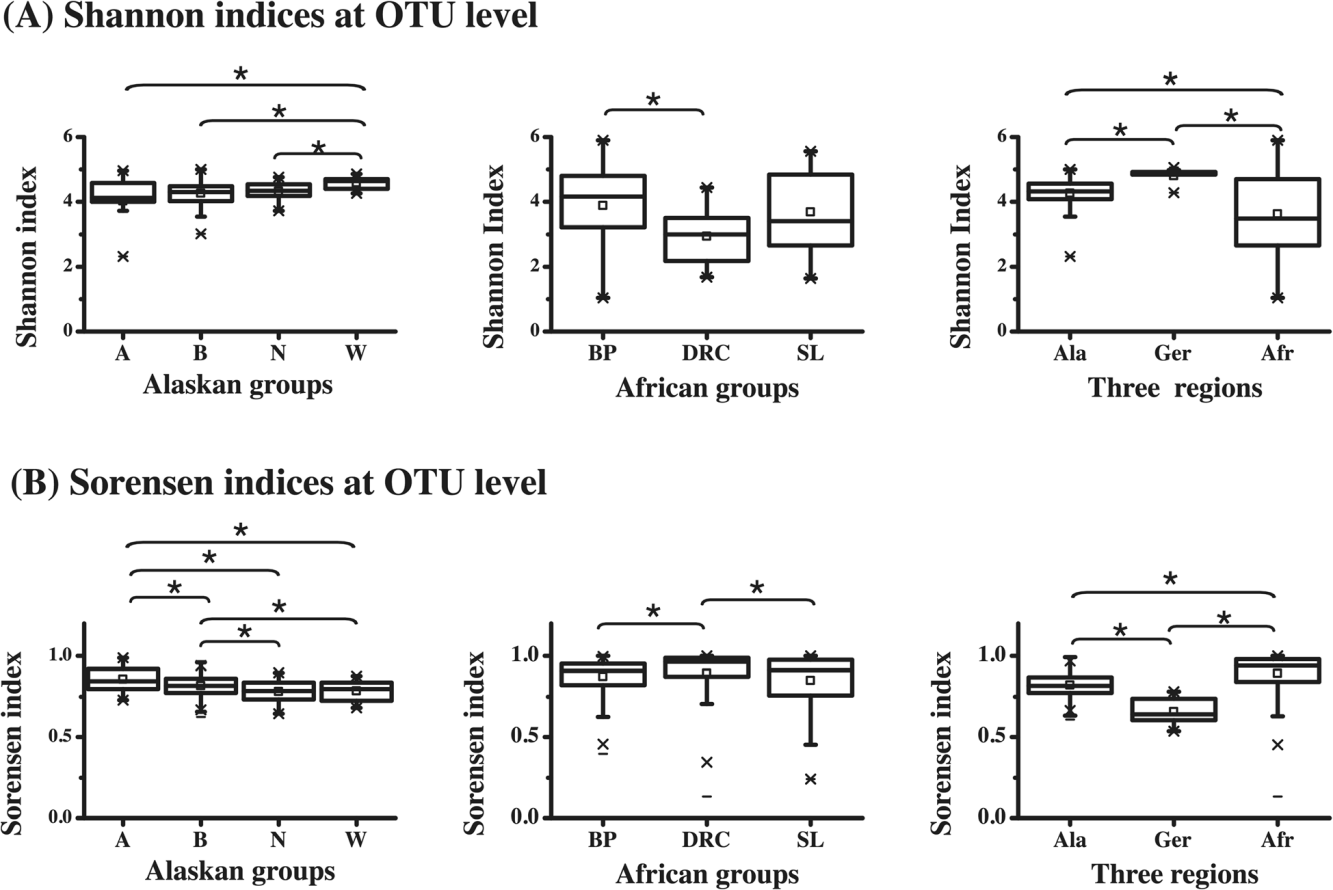
**Diversity analysis for four Alaskan groups, three African groups, and among the three continental regions based on the bacteria abundance distribution for each individual at OTU level. A**. Alpha diversity measured by Shannon indices and **B**. Beta diversity measured by Sørensen indices.

We also calculated the alpha and beta diversity at the OTU level (Figure 5). All populations showed higher alpha diversity values (average Shannon index = 4.02) than at the genus level (average Shannon index = 1.85), and similarly much higher beta diversity values (average Sorenson index = 0.88) than at the genus level (average Sorenson index = 0.60). However, the overall patterns of alpha vs. beta diversity values for each population are similar at the genus and OTU levels (Additional file 2: Figure S2 and Figure 5). The ANOSIM analysis based on OTUs confirmed the results based on genera (i.e. no significant differences among the four native Alaskan groups (P value = 0.2131) but significant differences between the native Alaskan group and both the Germans and the African group (P = 0.0131 and 0.0001, respectively).

### Core microbiome

**Figure 6:**
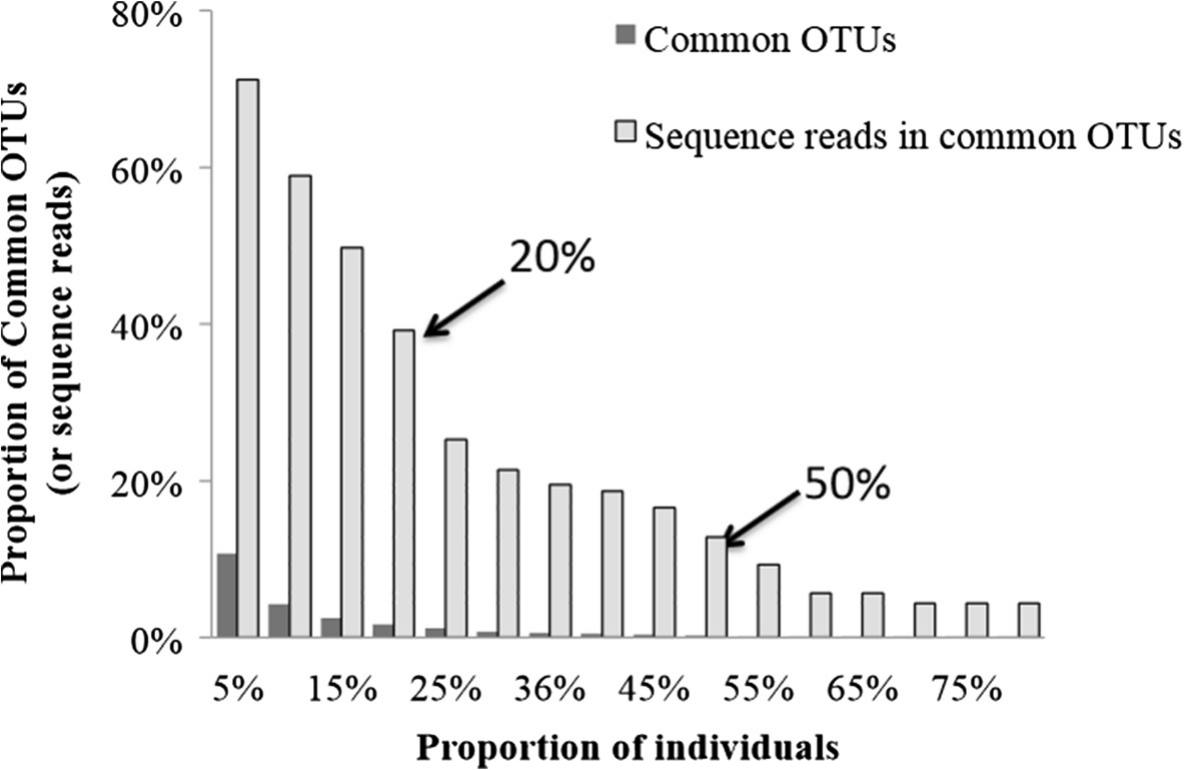
**Distribution of common OTUs and the total number of sequence reads accounted for by each OTU across the total of 152 individuals.** The dark bars represent the proportion of common OTUs among the total number of OTUs (6222 OTUs), and the light bars represent the proportions of sequence reads distributed in those common OTUs among the total number of sequences (72551 sequences).

In addition to analyzing the diversity of the human saliva microbiome, we also investigated the existence of a core microbiome in human saliva, i.e. a set of common OTUs shared across individuals from different environments. Figure 6 shows the distribution of shared OTUs across different percentages of the total number of individuals, and the corresponding percentage of the total sequences distributed in those shared OTUs. No OTUs are shared by more than 90% of the individuals; only 14 OTUs (0.23% of the total OTUs) were present in 50% of the individuals, and only 102 OTUs (1.64% of the total OTUs) were present in 20% of the individuals. However, the total number of sequences is significantly enriched in the shared OTUs: 9341 sequences (12.88% of the total sequences) were found in the OTUs shared by 50% of the individuals, and 28411 sequences (39.16% of the total sequences) were found in the OTUs shared by 20% of the individuals.

To determine if particular OTUs may be associated with particular environments, we investigated in more detail the patterns of shared and unique OTUs. Here, an OTU is considered to be exclusively shared if it is found in at least one member of each population within the continental groups compared. An OTU was considered unique, when it was exclusively present in one continental group but shared among all populations within this group. There are very few OTUs (0.16% - 0.22%) shared exclusively by two regional groups (Table 2), and the percentage of sequences distributed in the shared OTUs is also quite low (0.20% -0.66%). Conversely, there are 56 OTUs shared by all three continental groups (1.00%), with 20.92% of the total sequences distributed in those OTUs. These 56 OTUs were distributed over all major phyla except for TM7, and included the genera *Actinomyces*, *Fusobacterium*, *Lactobacillus*, *Haemophilus*, *Leptotrichia*, *Neisseria*, *Porphyromonas*, *Prevotella*, *Rothia*, *Streptococcus*, and *Veillonella* (abundance distribution see Additional file 9: Table S3). Additionally, we compared the taxa assignments of these 56 common OTUs with the results from the salivary core-microbiome identified from the Human Microbiome project (HMP) [29,30]. At the genus level, 10 of 11 assigned genera, except *Haemophilus*, from these 56 common OTUs were also detected in the HMP salivary core-microbiome (Additional file 9: Table S3). In contrast to the common OTUs, there are also very few OTUs that are unique to either Alaskans (0.23% OTUs with 0.71% sequences) or Germans (1.65% OTUs with 0.18% sequences), but many more OTUs unique to Africans (2.1% OTUs with 9.25% sequences), Table 2.

**Table 2:** **Number and percentage of OTUs and the sequences distributed in those OTUs that are either shared by different continental groups or unique to a single continental group**

		**OTUs**		**Sequences in those OTUs**	
		**Number**	**Percentage (%)**	**Number**	**Percentage (%)**
Shared OTUs	Alaskan-German	12	0.22	450	0.66
	Alaskan-African	9	0.16	285	0.42
	German-African	12	0.21	136	0.20
	3 Groups	56	1.00	14211	20.92
Unique OTUs	Alaskan	13	0.23	481	0.71
	German	92	1.65	121	0.18
	African	118	2.1	6285	9.25

### Influence of uneven sampling

The above results could potentially be biased because of the unequal number of sequence reads from each individual and unequal number of individuals from each population. Thus, to evaluate the effect of uneven sequencing and sampling, we re-performed all calculations based on randomly subsampling ~2500 reads from ~12 individuals from each population. As expected, the absolute number of observed genera and OTUs are decreased due to the reduced sampling reads (Additional file 10: Table S4). However, the major results from the comparisons among populations or groups did not change, e.g. the AMOVA analysis (Additional file 10: Table S4), rarefaction analysis (Additional file 11: Figure S7), UniFrac analysis (Additional file 12: Figure S8), and diversity analysis (Additional file 13: Figure S9). In another approach we separately subsampled 10 individuals from Alaskans, Germans, and Africans while also correcting for the unequal number of reads, i.e. we sampled ~2500 reads from each group. The corresponding results were similar to those based on the original number of individuals as can be seen in the AMOVA analysis (Additional file 10: Table S4), rarefaction analysis (Additional file 14: Figure S10), and diversity analysis (Additional file 15: Figure S11).

## Discussion

We have analyzed the salivary microbiome of human populations living in different geographic and climatic environments, including native Alaskans, Germans, and Africans. Although we lacked information to test for associations between variation in the salivary microbiome and demographic variables such as age or gender of the individuals, in a previous study of the saliva microbiome that encompassed a much larger geographic sampling, it was found that neither age nor gender of the individual influenced the variation in the saliva microbiome [21]. In addition, detailed examination of the oral health status or history of the donors was not carried out, nor was information available concerning dental hygiene practices of the donor; however, all donors were healthy and no donors were suffering from obvious oral lesions or diseases. Moreover, we do not detect consistent differences among groups in bacterial genera that have been previously shown to be associated with variation in dental hygiene (Figure 2), such as *Fusobacterium*, *Porphyromonas* and *Prevotella* [31]. Thus, the differences discussed below are more likely to reflect differences in geography, climate, and/or diet, rather than differences in oral health/hygiene (although the latter cannot be ruled out completely).

Although the four Alaskan groups are located in different regions (i.e. Atqasuk and Nuiqsut are in the inland of Alaska, while Barrow and Wainwright are located along the coast of Alaska, covering an overall distance of about 350 km), no significant differences among these four Alaskan groups were observed at both the genus and the OTU level. Therefore, geographic location and potentially different diets (the inland groups eat more caribou and less marine mammals than the coastal groups) did not play a crucial role in shaping the salivary microbiome composition of the four Alaskan groups. Conversely, the three African groups differed significantly, with the Batwa Pygmies (hunter-gatherer lifestyle) showing significantly higher saliva microbiome diversity compared to the other two African groups (farmer lifestyle). Hence, lifestyle, geography and/or diet also seem to play a role in shaping the salivary microbiome in the African groups. For comparison, we also analyzed the salivary microbiome composition of a German group, representing an intermediate geographic location with moderate climatic conditions. Interestingly, the salivary microbiome of the German group showed a high similarity with the Alaskan group at the genus level, while at the OTU level distinct differences could be observed. Overall, the salivary microbiome of the human populations from the northern continents were of comparable similarity, but differed strongly from the African groups at both the genus and OTU level. This finding corroborates our previous study, which showed an association between UniFrac distances and the geographic distance of analyzed individuals from the equator [21].

Further differences among the salivary microbiomes of the three geographic regions are revealed when looking at alpha and beta diversity. Differences were more pronounced at the OTU-level than at the genus-level. Alpha diversity was highest for the German group and lowest for the African group, while the opposite was true for beta diversity (Additional file 2; Figure S2 and Figure 5), which is in agreement with the AMOVA analysis (Table 1). One potential explanation for the differences in alpha diversity could be the diversity of the diet. The diet of the German individuals probably encompasses a wider variety of substances (especially a multitude of different carbohydrates) than the diet of the native Alaskans or Africans, and those nutrients have an affect on the ecology of the mouth [32]. We therefore speculate that a rich buffet of different foodstuffs may provide a more complex array of substrates and thus ultimately allow a higher number of bacterial species (including low-abundant members) to thrive in the oral cavity, which would explain the differences in alpha-diversity observed in our study. Another explanation could be population density, which is higher in Germany than in the other regions, providing more opportunities for bacteria to be spread among individuals. High beta diversity reflects the heterogeneity of the samples, which in turn might be the result of the size and diversity of the sampled area (e.g. the German individuals reflect a more homogenous group coming from a fairly restricted area, while the Alaskan and African groups originated from far bigger geographic areas). Large areas are environmentally more heterogeneous than small areas and as a consequence the overall diversity of observed bacteria might be higher. Hence, despite relatively low diversity within individuals, the overall number of bacterial taxa may be higher in more geographically dispersed groups, as was observed for the African group (Table 1).

Studies on bacterial interaction within human ecosystems are still in their infancy, however they address important and challenging open questions regarding the human microbiome: namely, how is human health affected by the trophic interdependencies and disturbances among bacterial taxa? Here we used partial correlation analysis to construct the interaction network for each regional group (Alaskans, Germans, and Africans) based on genera. Interestingly, the resulting interactions were mostly unique to one particular human group. Although currently largely speculative in nature, these interactions may reflect the versatility of microbial trophic webs within humans [33]. As such these interactions might provide the basis for a better distinction of different human populations, which otherwise may not be distinguishable by the mere composition of their microbiota. Even at the phylum level a distinction was observed, since the African group showed a lower number of negative interactions, while the proportion of positive interactions were largely consistent among the human groups. Partial correlation analysis also showed that the Alaskan group and the German group shared the highest number of common interactions between genera, corroborating the overall result of this study. The network of 40-shared interactions (between at least two geographical regions) included also the recognized association of early, middle and late oral colonizers (*Porphyromonas*, *Fusobacterium* and *Aggregatibacter*, respectively [34]. Hence, the associations known from *in-vitro* experiments seem to stably exist in oral ecological niches across different ethnic hosts and/or different geographic environments. Furthermore four pairs of positive interactions were identified present in all human populations. Apparently, the involved genera (i.e. *Actinomyces*, *Gemella*, *Granulicatella*, *Veillonella, Haemophilus, Capnocytophaga* and TM 7) are not only permanently prevalent (though with different abundances, as can be seen in Figure 1), but may reflect fundamental bacterial relationships in the human oral cavity (regardless of ethnicity or geographic origin). As such those genera may have a key role in human oral health or disease. This assumption is further warranted by the recognized central position of early colonizing *Veillonella* that emerges in saliva as a critical genus guiding the development of multispecies communities [34], and by the emerging key role of members of the phylum TM 7 in the salivary microbiome [35].

A recent comprehensive study examined microbial co-occurrences or co-exclusion in different human body sites [36]. However, comparisons with our study are not straightforward, as saliva samples made up only 6% of their samples and microbial relationships are presented largely at the family or class level. There is only one reported positive association at the genus level in saliva, namely between *Aggregatibacter* and *Capnocytophaga*, which we also observe in the African individuals. Otherwise, it is notable that most bacterial interactions were found in the oral cavity compared to other body sites [35], which may explain the relatively high number of interactions we found in our study.

Despite the overall higher similarity between the Alaskan and German salivary microbiome, they did not share substantially more OTUs (i.e. 12), than the Alaskan and African group or the German and African group (9, and 12, respectively). Hence, resemblance of salivary microbiomes between human populations rather occurs at higher taxonomic ranks and in how the microbiomes are structured (e.g. beta and alpha diversity). In addition, the relatively low number of OTUs shared among all continental groups (i.e. 56 OTUs accounting for 21% of all reads) provides at best limited support for the concept of a core human saliva microbiome at the 97% OTU level. Clearly, a core human microbiome (if truly existing) can be defined in more complex ways, depending on the ecological questions addressed [37]. However, it can be concluded that these 56 common OTUs probably represent key organisms that are important for sustaining the salivary microbial ecosystems in at least some humans.

Interestingly the number of unique OTUs (i.e. those found only in one continental group but shared among all populations within this group) was highest in Africa, although the African group had the highest heterogeneity (according to the results of the beta diversity and AMOVA analyses). Most of these African specific OTUs are enriched in *Enterobacteriaceae* (i.e. *Enterobacter, Klebsiella and Escherichia*). In line with our previous observations [21,23], members of the family *Enterobactericeae* seem to be a consistent signature that distinguishes the salivary microbiome of African populations from other worldwide geographical regions. The reason for the high prevalence and abundance of *Enterobacteriaceae* in African populations remains currently unknown; knowledge of precise species would help elucidate the source of enterobacterial colonization (e.g. uptake of free-living species from plants vs. introduction through consumption of fecal-contaminated food or water). Besides diet or drinking water, we speculate that outdoor temperature is a hitherto unrecognized contributing factors that enhances/promotes the oral colonization of *Enterobacteriaceae* in Africans. First, members of *Enterobacteriaceae* grow best at temperatures beyond 37 degrees Celsius (e.g. the optimum growth rate of *Escherichia coli* and *Enterobacter sakazakii* is 40.85 and 39.4 degrees Celsius, respectively [38,39]. Second, increased temperature by seasonal changes has been shown to be associated with increased human colonization by *Enterobacteriaceae,* as deduced from retrospective studies on Gram-negative bacterial bloodstream infections [40,41]. Third, outdoor temperature affects the oral temperature [42,43] and it is plausible to assume that the oral temperature of African individuals is at least slightly elevated compared to individuals from other climatic zones and therefore more amenable for enterobacterial growth. In addition to the temperature level itself, the constancy of the oral temperature may influence bacterial growth. It is conceivable that sudden drops or rise in oral temperature occur within Alaskan and German individuals (especially during wintertime) when leaving or entering heated houses, which clearly is not the case for individuals with close proximity to the equator.

We are aware that the unequal number of sequence reads from each individual and unequal number of individuals from each population could bias some of our results based on absolute observations, such as unique/shared taxa. However, after subsampling individuals and sequence reads, our main conclusions based on analyses of relative abundances (phylogenetic analysis, clustering analysis, and diversity analysis) did not change substantially. Thus, the uneven sampling is not responsible for the major results of the analyses.

## Conclusions

In conclusion, we have shown that human populations from different geographic and climatic areas exhibit differences in their salivary microbiome, the reasons of which (e.g. differential lifestyles including diet and/or host genetics and physiology including the immune system) remain to be elucidated. This knowledge is vital for a proper definition of the global salivary microbiome in health and disease. Further studies including other environments and geographic regions are therefore warranted.

## Methods

### Ethics statement

All participants provided written informed consent, and this study was approved by the Northwestern University Institutional Review Board and the Ethics Committee of the University of Leipzig Medical Faculty.

### Samples and DNA extraction

Saliva samples were collected from four native Alaskan communities from Atqasuk (14 samples), Barrow (40 samples), Nuiqsut (13 samples) and Wainwright (9 samples) (Figure S1). Samples were also obtained from 10 individuals living in or nearby Leipzig, Germany. DNA was extracted from the German samples as described previously [44], and from the native Alaskan samples with the Oragene kit, following the manufacturer’s directions. For comparison, we included published data [23], generated with similar methods, for three groups from Africa (Democratic republic of Congo [DRC, 15 samples], Sierra Leone [SL, 13 samples] and Batwa Pygmies from Uganda [BP, 38 samples]). For sample collection, volunteers spit up to 2 mL of saliva into tubes containing 2 mL lysis buffer [44]. While the oral health of donors at the time of sampling was not investigated in detail, no human donor was suffering from obvious oral lesions or severe dental decay, and to the best of our knowledge no human was being treated with antibiotics at the time of sampling. The age of the human donors ranged from 20–40 years.

### PCR amplification of the microbial 16S rRNA gene

We amplified a region of the microbial 16S rRNA gene containing variable segments V1 and V2, which were previously shown to be more informative than other regions of the 16S rRNA gene in terms of the number of phylotypes detected [45]. We used the forward primer for V1 and the reverse primer for V2 [45], which together amplify a ~350 bp PCR product containing V1 and V2. Here, more detailed extraction protocols and PCR primers could be found in the previous published work [45], and the same extraction and PCR condition were used for all samples.

### Sequencing on the genome sequencer FLX platform

The PCR products were processed for parallel-tagged sequencing on the Genome Sequencer FLX platform, as described previously [21,46]. Sample-specific barcode sequences were ligated to the PCR products, and DNA concentrations were assessed on an Mx3005P™ (Stratagene). Samples were then pooled in equimolar ratios to a total DNA amount of 440 ng. The pooled library was subsequently amplified in PCR-mixture-in-oil emulsions and sequenced on one lane of a 4-lane Pico Titer Plate on a Genome Sequencer FLX/454 Life Sciences sequencer (Branford CT), according to the manufacturer’s protocol.

### Data analysis

The initial sequence reads were filtered to remove sequence reads containing two or more different tags, no tags, primers in the middle of sequence reads, or without a primer sequence. These sequence reads have been deposited in Genebank Sequence Read Archive (SRA) SRP028342. The aligned sequences used in the analyses are available from the authors upon request. Quality filtering of the raw sequence reads was performed with the AmpliconNoise pipeline [25], and the chimeras were identified by the Mallard [47] program by using a quantile value of 95% as the threshold for removing outliers. The filtered sequences were assigned to different genera by the Classifier approach [26] in the Ribosomal Database Project (RDP) database [48]. Diversity statistics and apportionment of variation based on the frequency distribution of genera within and between individuals were calculated with Arlequin 3.5 [49]. Spearman’s rank correlation coefficients were calculated with the R package. Diversity (dissimilarity) analysis, Shannon-Weaver index (alpha diversity), Sørensen index (beta diversity) were calculated by the “vegan” package in R, and the significances of diversity distributions between groups were implemented by the Mann–Whitney u test. The dissimilarity tests among groups (ANOSIM) were also implemented by the “vegan” package in R. Rarefaction analysis was carried out using the Resampling Rarefaction 1.3 software (http://strata.uga.edu/software/). For the UniFrac analysis, the sequences were aligned with the Infernal 1.0 program [50] and a phylogenetic tree was constructed under a generalized time reversible (GTR) model with the FastTree software [51]. Fast UniFrac [52] was then used to compare the microbial communities, compute the distance matrix, and generate the cluster tree. The OTUs networks were constructed from the sequences aligned with Infernal 1.0 by using tools provided by the RDP website to first cluster all sequences that were 97% or more similar (based on a minimum overlap of 25 bases) into OTUs (to account for sequencing errors). We then used the Cytoscape 2.8 software [53] to generate and visualize the networks. Briefly, each individual is considered a Source node and each OTU is a Target node. Target nodes were linked to Source nodes in a bipartite network, with connections between Sources and Targets modeled as springs; both Source and Target nodes are placed in such a way as to minimize the forces across the network. The partial correlation network was constructed by “GeneNet” package in R [54]. The partial correlation coefficients were calculated from the abundance frequency of the 123 genera for the 152 individuals and the significance of correlations was estimated by permutation. We used an FDR (false discovery rate) of 0.01 as a cutoff to choose the top-ranked correlations among genera to build the connection network. For simplicity, we only displayed the connections among the 28 most common genera (i.e. those with a frequency >0.5% in either the Alaskan, German or African group). Additionally, the phylogenetic tree in Figure 1 was implemented in the ARB program package [55] using the Jukes-Cantor correction.

## Additional files

Additional file 1: Figure S1. Map of sampling locations and pie charts showing the frequencies of the distribution of microbial genera detected in 4 native Alaskan groups. Additional file 2: Figure S2. Alpha and beta diversity analysis for four Alaskan groups at the genus level. The stars between two groups indicate significant differences, based on Mann–Whitney U tests. Additional file 3: Figure S3. Heat plot of the abundance of each bacterial genus in each individual. Each numbered column corresponds to a genus, and each row is an individual saliva sample. Additional file 4: Table S1. Frequency differences in genera abundance. Additional file 5: Figure S4. Pairwise correlation matrix between individuals calculated from bacteria abundance at the genus level. Additional file 6: Table S2. Number of connections from the partial correlation network assigned to different phyla. Additional file 7: Figure S5. Comparison of interactions at the phylum level. Additional file 8: Figure S6. Pairwise correlation matrix between individuals calculated from the frequency abundance at the OTU level. Additional file 9: Table S3. Comparison of 56 core OTUs with published results from HMP. Additional file 10: Table S4. Results from subsampling. Additional file 11: Figure S7. Comparison of the rarefaction analysis from original and subsampled reads. Additional file 12: Figure S8. Comparison of the UniFrac analysis from original and subsampled reads. Additional file 13: Figure S9. Comparison of the alpha- and beta-diversity analysis from original and subsampled reads. Additional file 14: Figure S10. Rarefaction analysis by subsampling 10 individuals from each group. Additional file 15: Figure S11. Diversity analysis by subsampling 10 individuals from each group.
