# Supplementary figures and images for: Comparative analysis of the human saliva microbiome from different climate zones: Alaska, Germany, and Africa
Source: BMC Microbiol. 2014 Dec 17;14:316. doi: 10.1186/s12866-014-0316-1 (PMC4272767; doi:10.1186/s12866-014-0316-1)

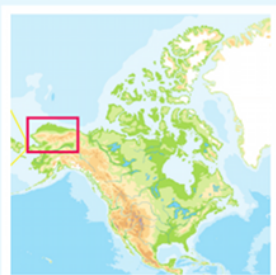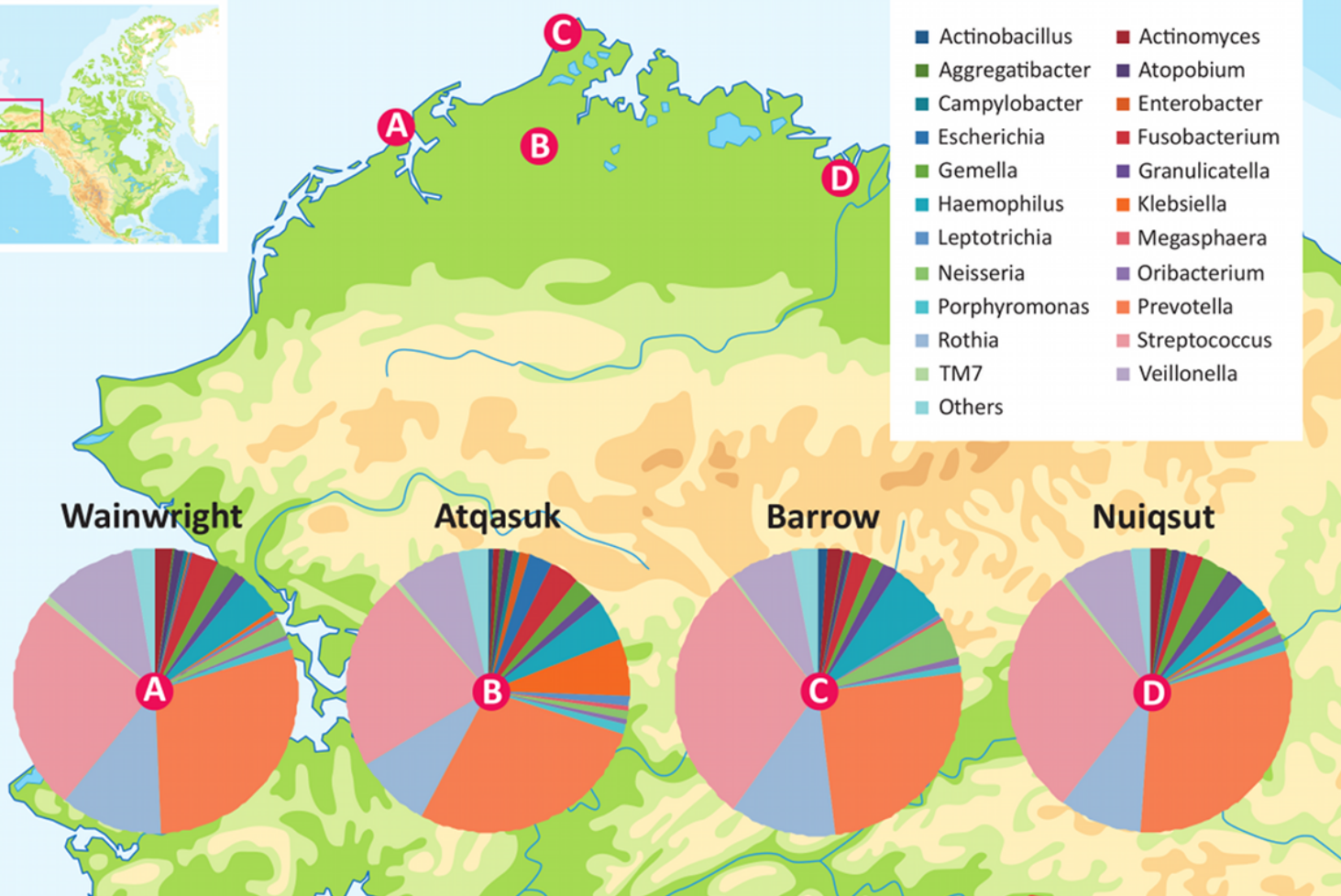

Supplement: Additional file 1: Figure S1. — Map of sampling locations and pie charts showing the frequencies of the distribution of microbial genera detected in 4 native Alaskan groups. [file 12866_2014_316_MOESM1_ESM.pdf]

## (A) Shannon indices at genus level

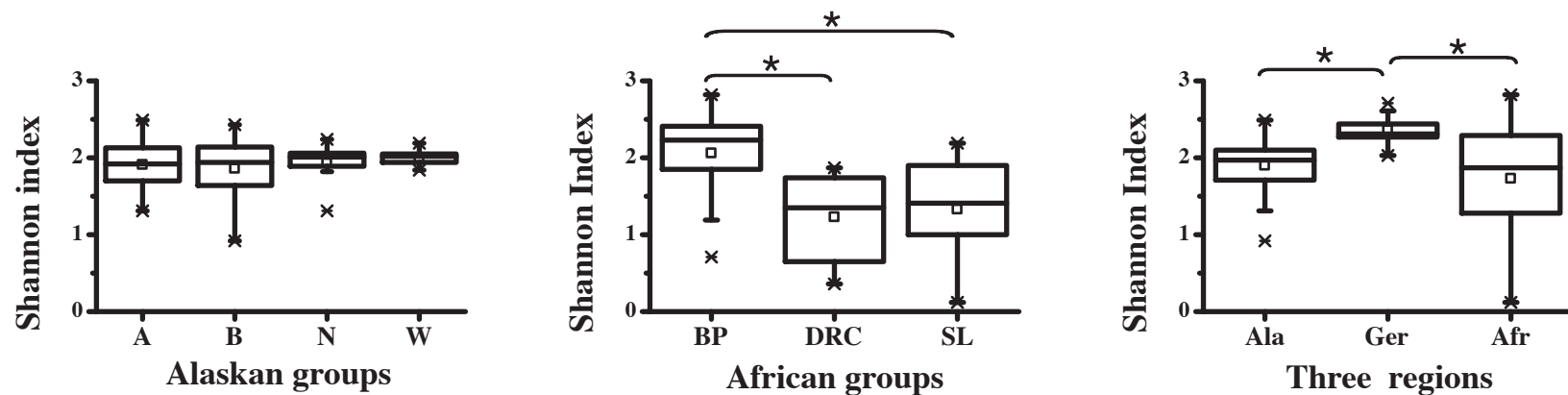

## (B) Sorensen indices at genus level

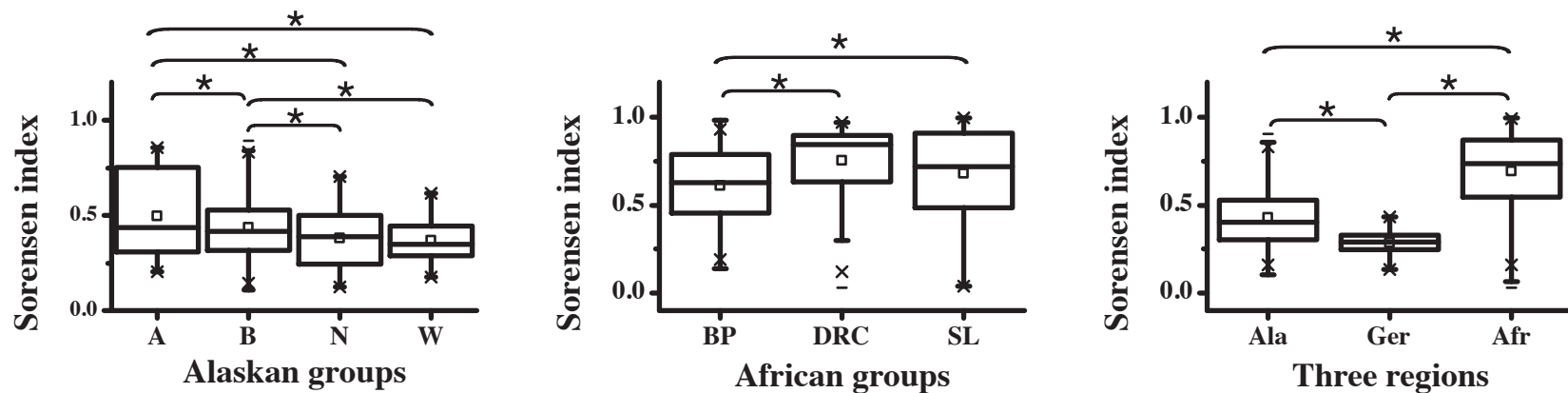

Supplement: Additional file 2: Figure S2. — Alpha and beta diversity analysis for four Alaskan groups at the genus level. The stars between two groups indicate significant differences, based on Mann–Whitney U tests. [file 12866_2014_316_MOESM2_ESM.pdf]

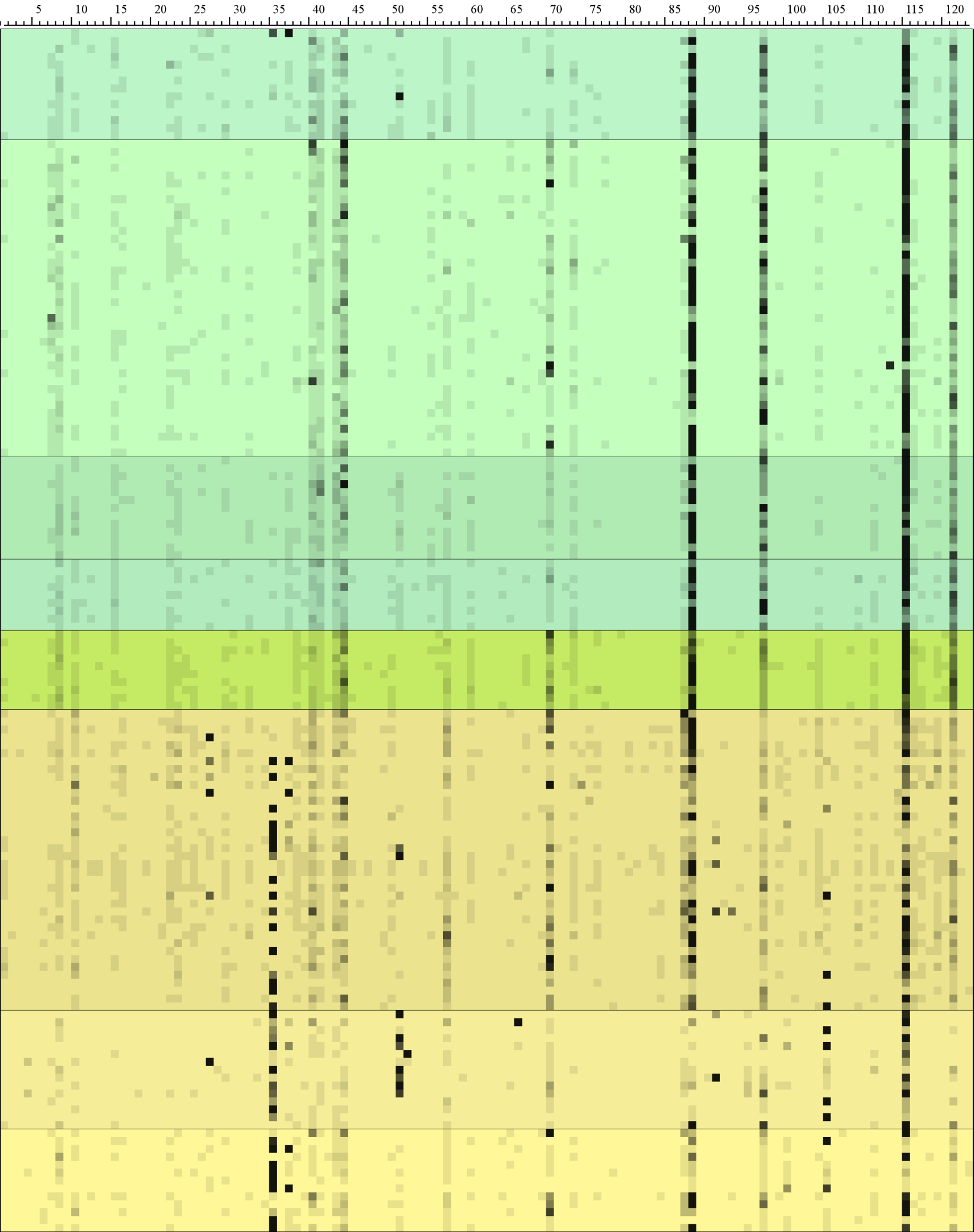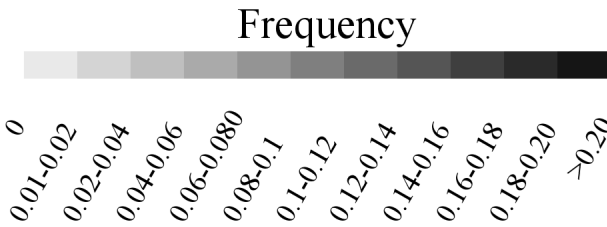

Supplement: Additional file 3: Figure S3. — Heat plot of the abundance of each bacterial genus in each individual. Each numbered column corresponds to a genus, and each row is an individual saliva sample. [file 12866_2014_316_MOESM3_ESM.pdf]

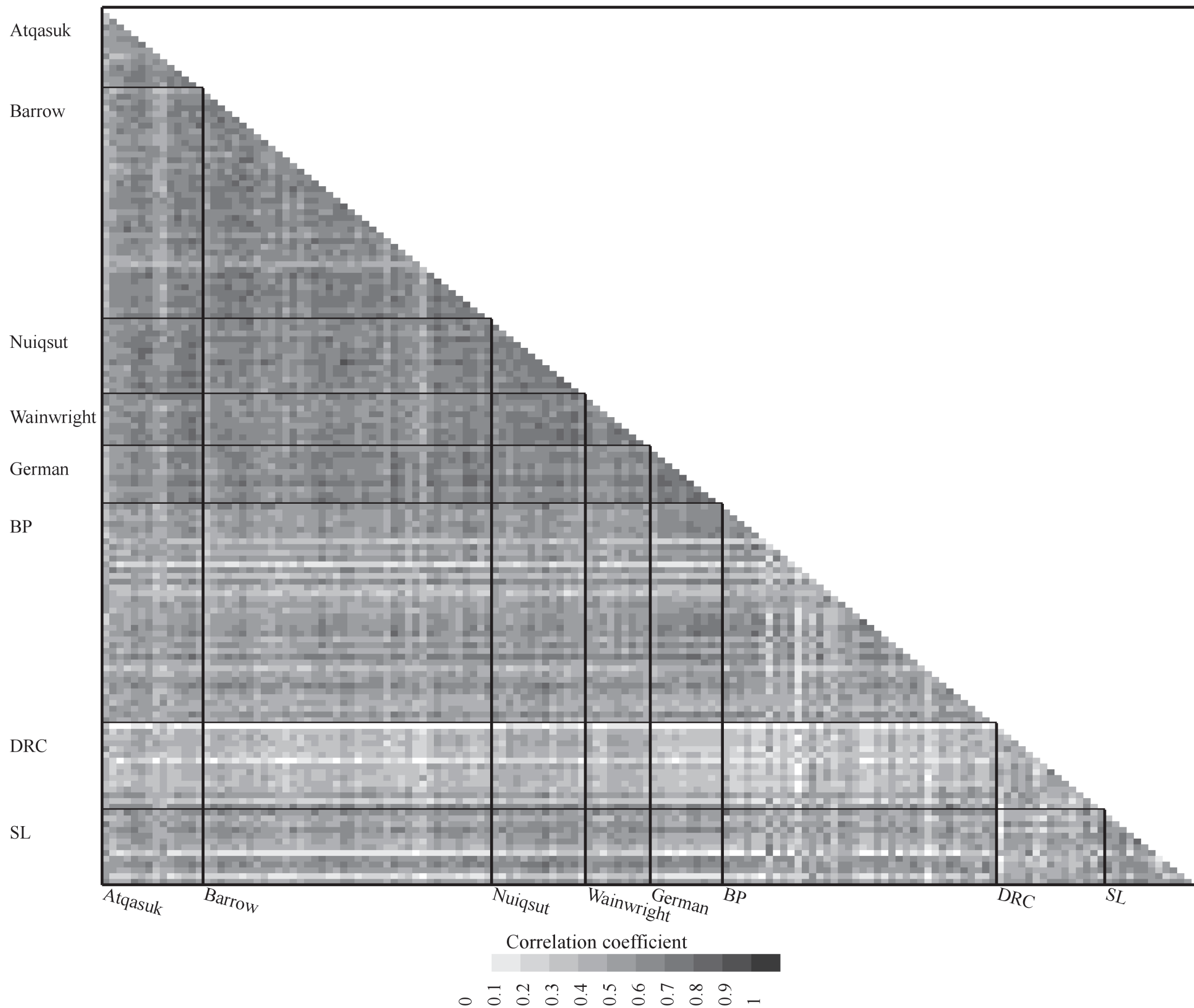

Supplement: Additional file 5: Figure S4. — Pairwise correlation matrix between individuals calculated from bacteria abundance at the genus level. [file 12866_2014_316_MOESM5_ESM.pdf]

## Positive minus negative interactions

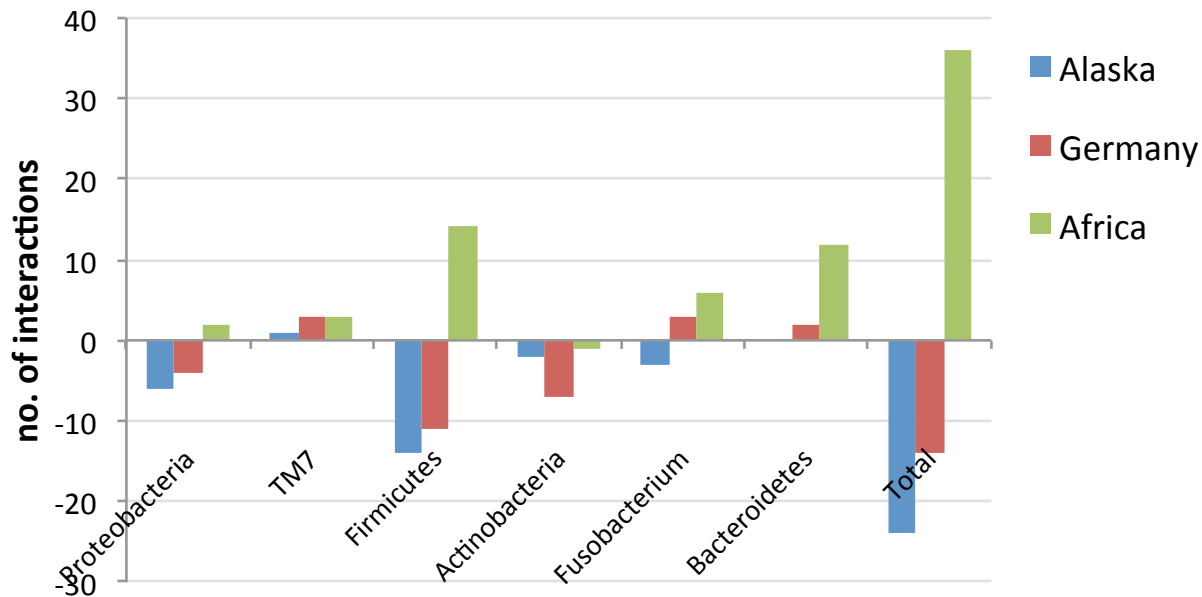

Supplement: Additional file 7: Figure S5. — Comparison of interactions at the phylum level. [file 12866_2014_316_MOESM7_ESM.pdf]

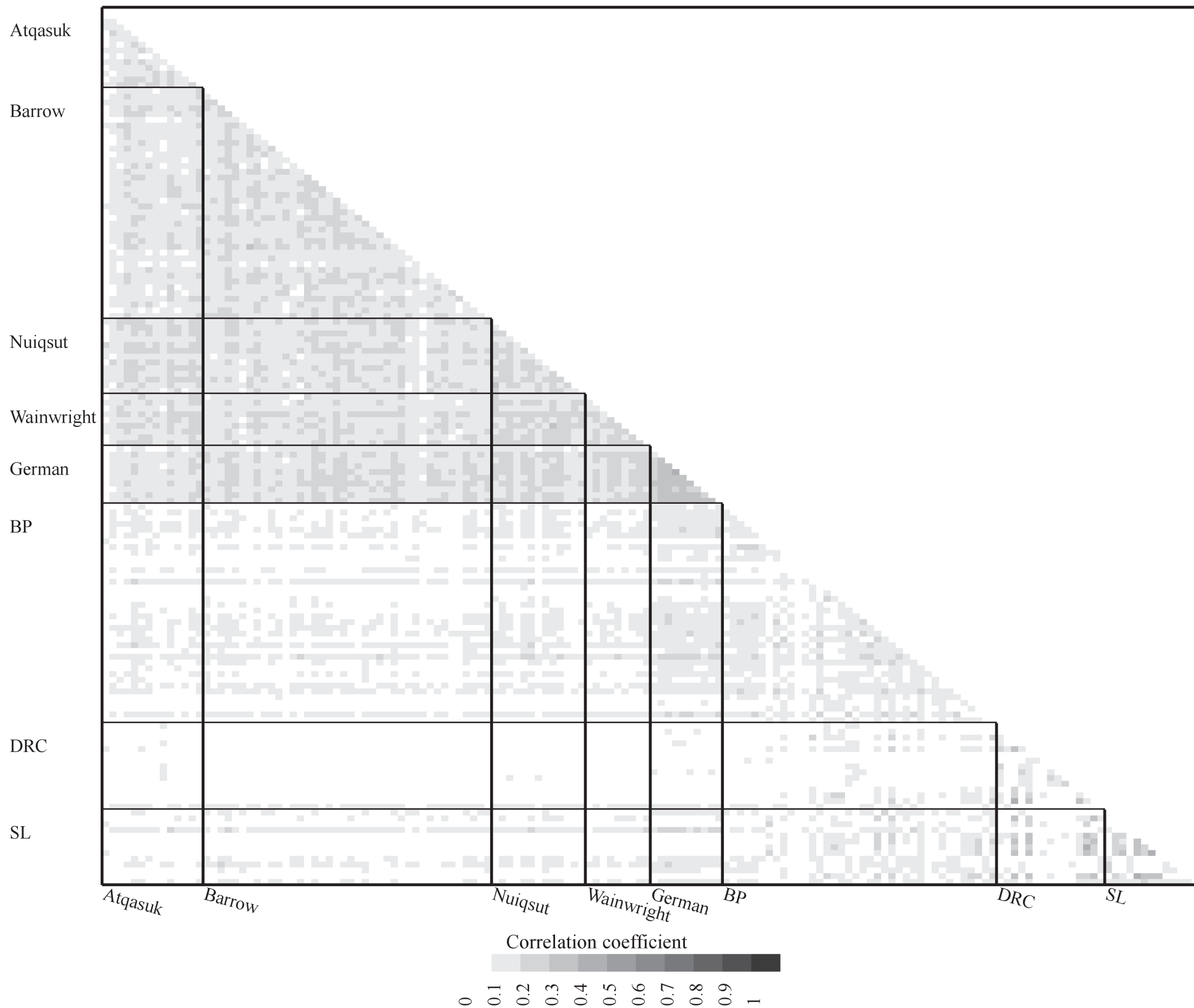

Supplement: Additional file 8: Figure S6. — Pairwise correlation matrix between individuals calculated from the frequency abundance at the OTU level. [file 12866_2014_316_MOESM8_ESM.pdf]

**A**

### Original sequenced reads

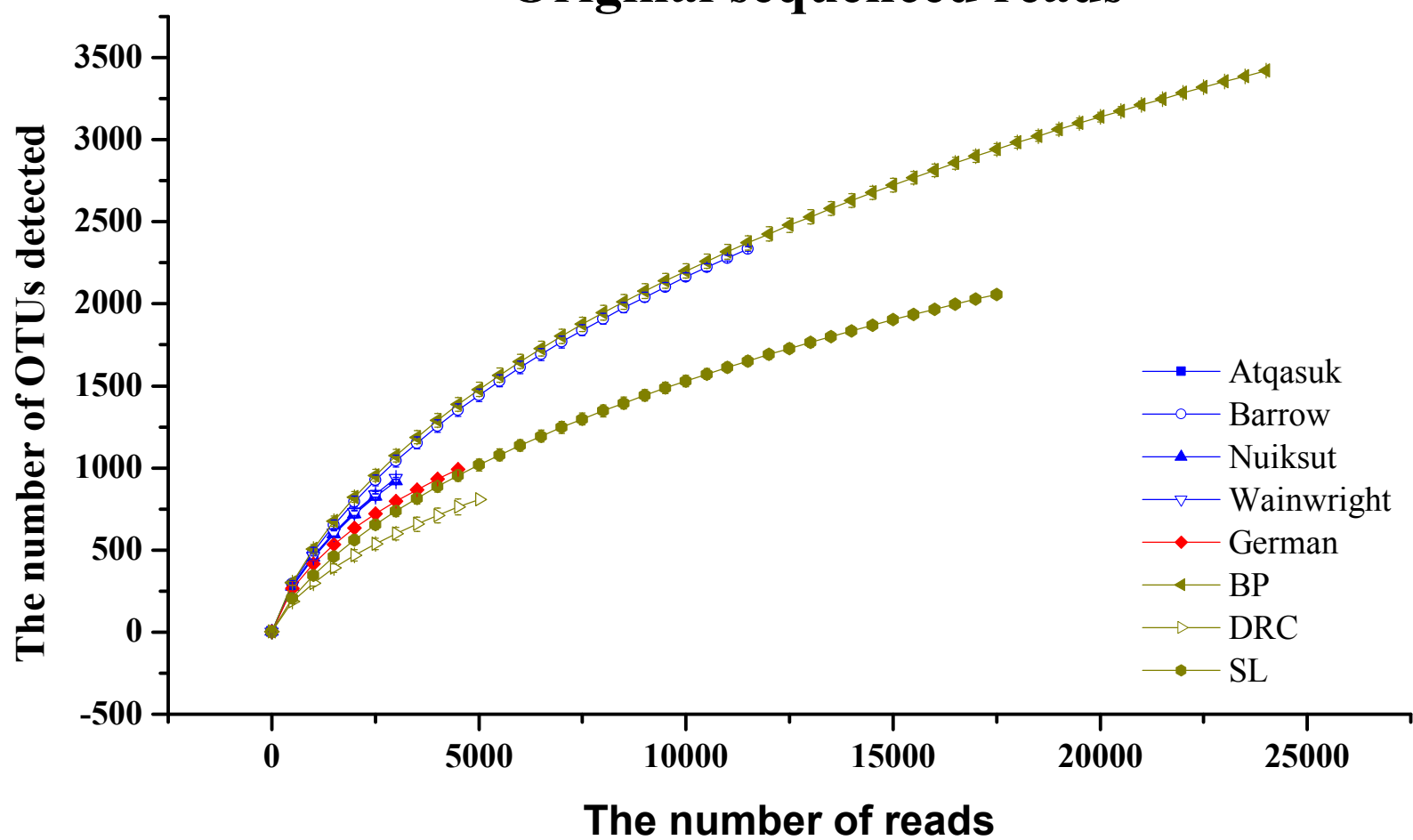**B**

### Randomly subsample ~2500 reads from each population

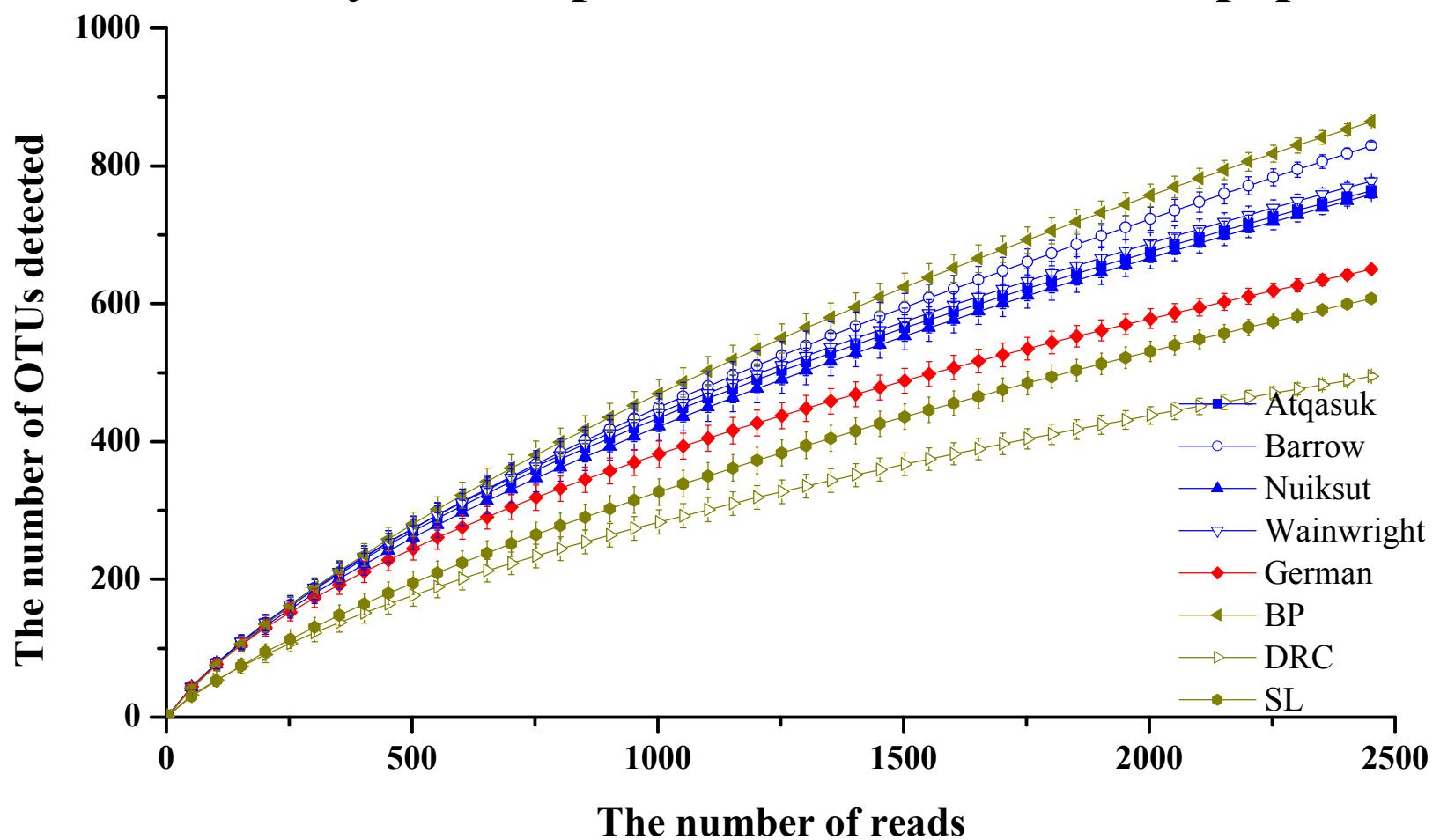

Supplement: Additional file 11: Figure S7. — Comparison of the rarefaction analysis from original and subsampled reads. [file 12866_2014_316_MOESM11_ESM.pdf]

# Original sequenced reads

A

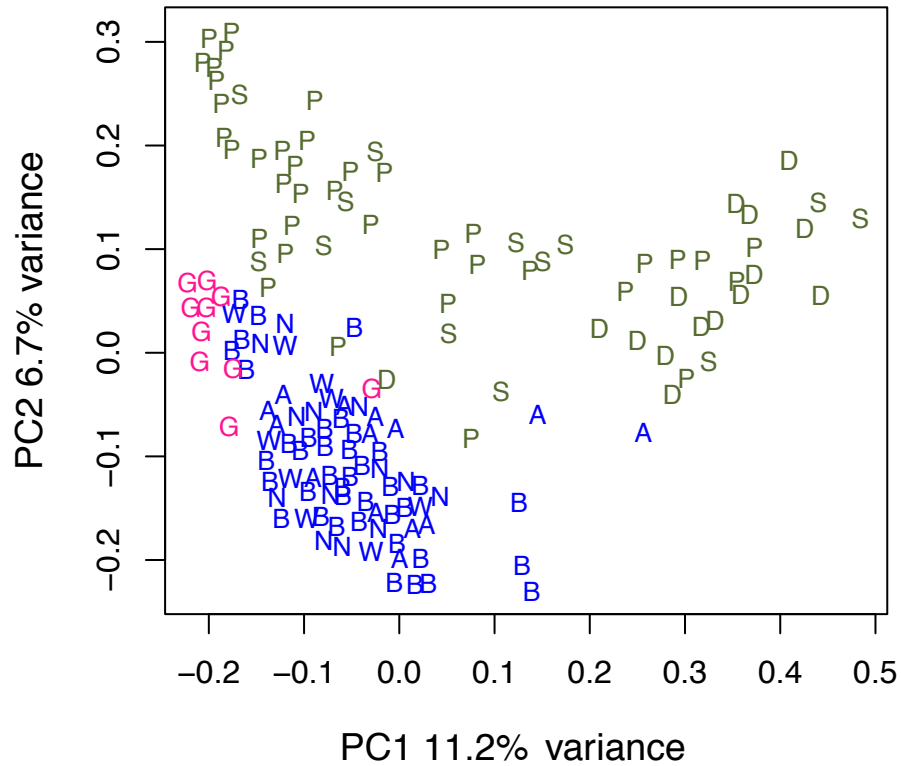

B

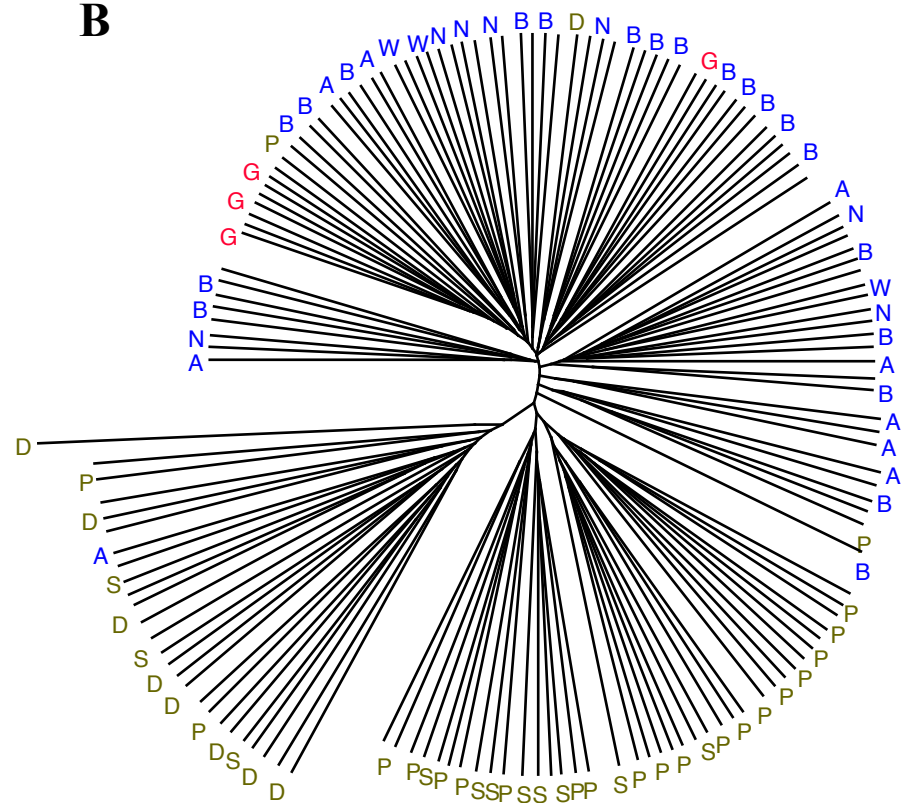

## Randomly Subsample ~2500 reads from each population

C

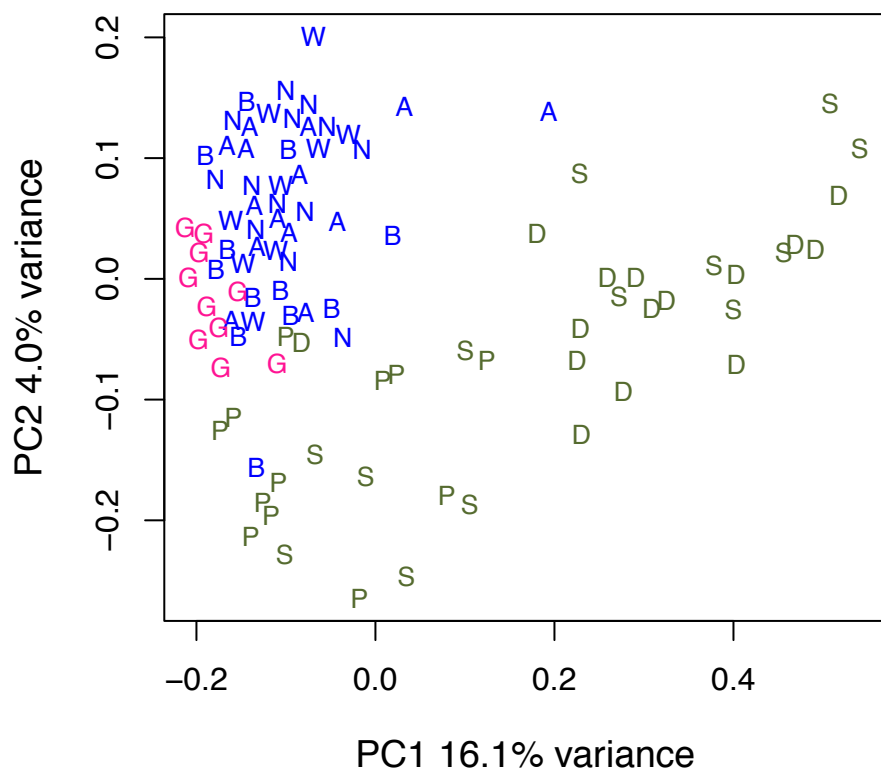

D

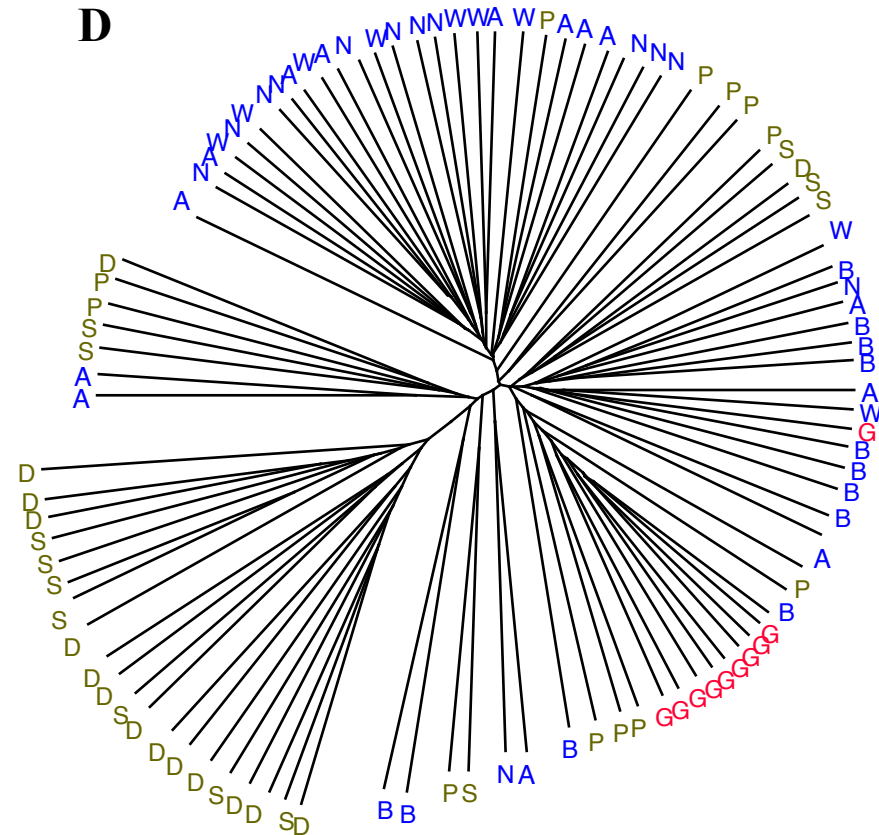

Supplement: Additional file 12: Figure S8. — Comparison of the UniFrac analysis from original and subsampled reads. [file 12866_2014_316_MOESM12_ESM.pdf]

Randomly subsample 10 individuals with ~2500 reads from each group

**A**

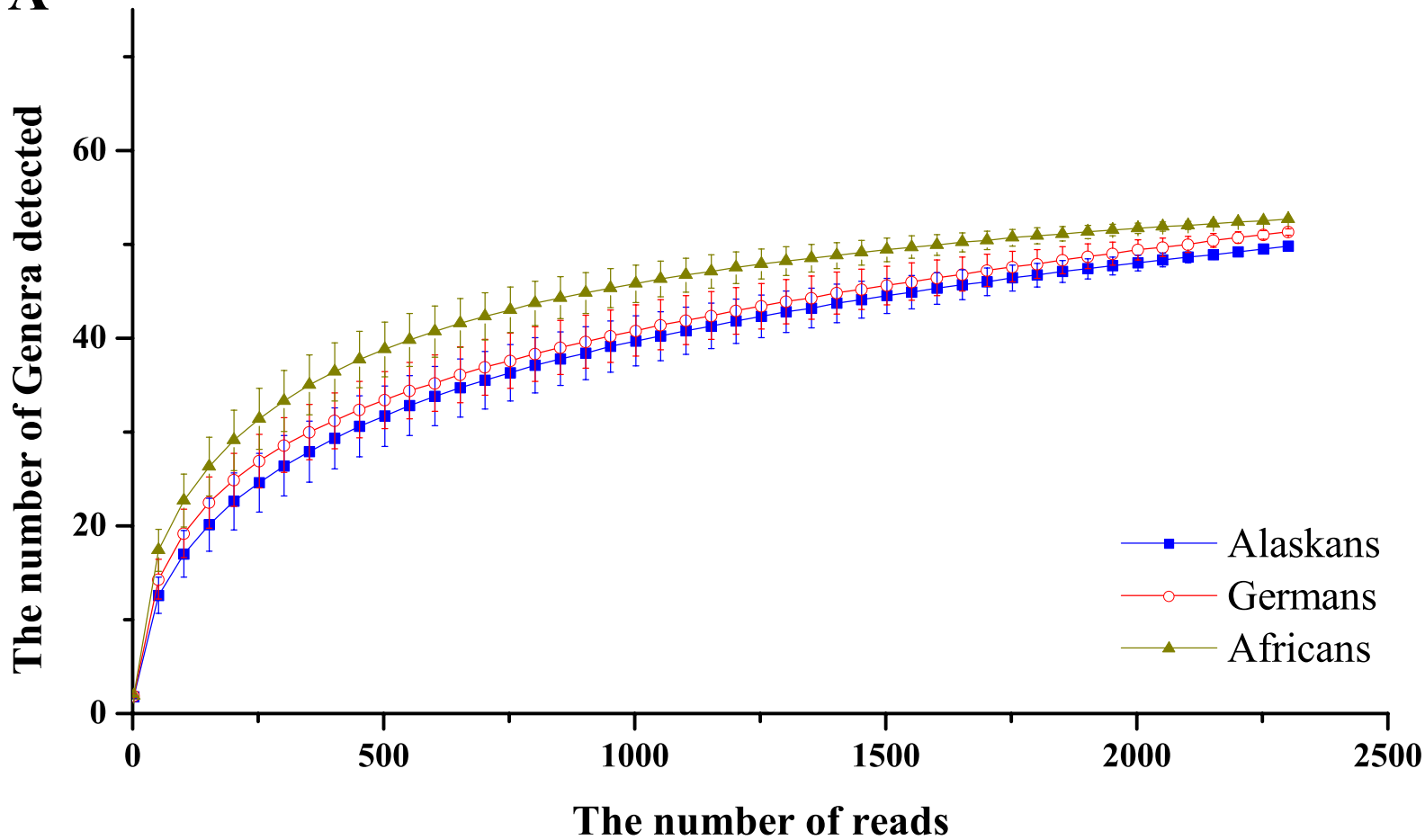

**B**

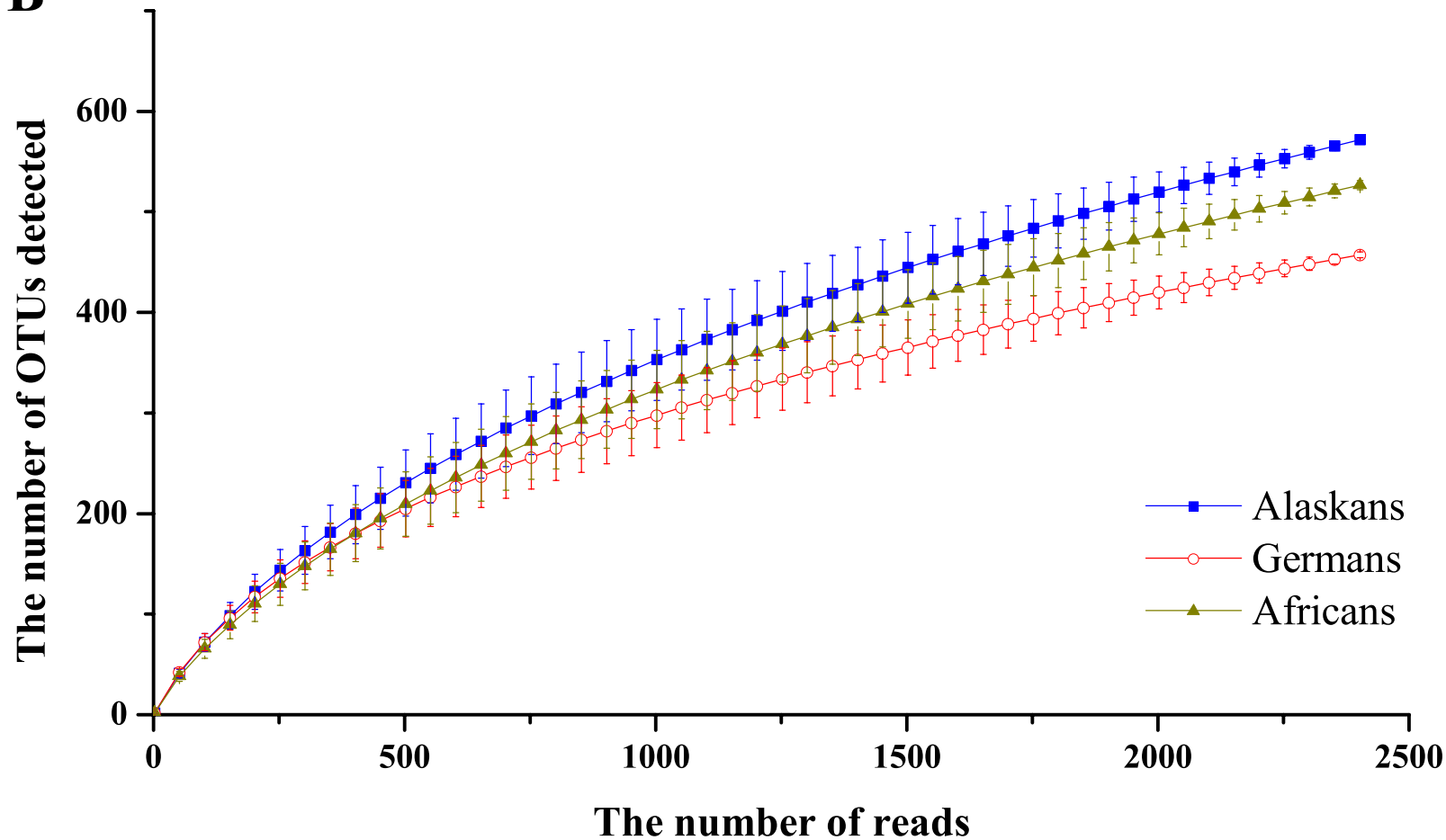

Supplement: Additional file 14: Figure S10. — Rarefaction analysis by subsampling 10 individuals from each group. [file 12866_2014_316_MOESM14_ESM.pdf]
