# Supplementary material for: Comparative analysis of the human saliva microbiome from different climate zones: Alaska, Germany, and Africa
Source: BMC Microbiol. 2014 Dec 17;14:316. doi: 10.1186/s12866-014-0316-1 (PMC4272767; doi:10.1186/s12866-014-0316-1)
Supplement: Additional file 4: Table S1. — Frequency differences in genera abundance. [file 12866_2014_316_MOESM4_ESM.pdf]

Table S1-a. Genera abundance difference among 4 Alaskan groups
*Here we take P value< 0.0005 as significant threshold, and only calculated for common genera which means abundance frequency > 0.5% in at least one group

Genera	Frequency				Significant (P value)
	Atqasuk	Barrow	Nuiksut	Wainwright	
Actinobacillus	0.005	0.010	0.001	0.001	0.0000*
Actinomyces	0.011	0.017	0.017	0.019	**0.0989**
Aggregatibacter	0.004	0.004	0.006	0.004	**0.3344**
Atopobium	0.010	0.003	0.008	0.011	0.0000*
Campylobacter	0.006	0.004	0.002	0.004	**0.3048**
Enterobacter	0.011	0.000	0.001	0.001	0.0000*
Escherichia	0.028	0.000	0.005	0.005	0.0000*
Fusobacterium	0.035	0.022	0.020	0.029	**0.0005**
Gemella	0.022	0.014	0.030	0.024	0.0000*
Granulicatella	0.015	0.017	0.021	0.013	**0.0796**
Haemophilus	0.046	0.066	0.039	0.042	0.0000*
Klebsiella	0.067	0.000	0.010	0.008	0.0000*
Leptotrichia	0.010	0.005	0.006	0.005	**0.0219**
Megasphaera	0.005	0.003	0.007	0.004	**0.0235**
Neisseria	0.013	0.046	0.014	0.020	0.0000*
Oribacterium	0.004	0.008	0.007	0.004	**0.0312**
Porphyromonas	0.012	0.010	0.012	0.010	**0.7502**
Prevotella	0.276	0.253	0.307	0.297	0.0000*
Rothia	0.087	0.121	0.097	0.115	0.0000*
Streptococcus	0.221	0.296	0.281	0.242	0.0000*
TM7_genera_incertae_sedis	0.006	0.004	0.007	0.006	**0.2026**
Veillonella	0.074	0.070	0.080	0.109	0.0000*

Table S1-b. Genera abundance difference between Alaskans, Germans, and Africans

*Here we take P value< 0.0005 as significant threshold, and only calculated for common genera which means abundance frequency > 0.5% in at least one group

Genera	Frequency				Significant (P value)		
	Alaskan	German	African	3 groups	Alaskans vs. Germans	Alaskans vs. Africans	Germans vs. Africans
Actinobacillus	0.0066	0.0049	0.0011	0.0000*	**0.2253**	0.0000*	0.0000*
Actinomyces	0.0162	0.0320	0.0074	0.0000*	0.0000*	0.0000*	0.0000*
Aggregatibacter	0.0043	0.0078	0.0106	0.0000*	**0.0034**	0.0000*	**0.0802**
Atopobium	0.0058	0.0066	0.0011	0.0000*	**0.5607**	0.0000*	0.0000*
Campylobacter	0.0036	0.0080	0.0043	**0.0005**	0.0001*	**0.2327**	0.0008*
Capnocytophaga	0.0028	0.0026	0.0058	0.0000*	**0.7857**	0.0000*	**0.0074**
Citrobacter	0.0005	0.0000	0.0188	0.0000*	**0.1318**	0.0000*	0.0000*
Enterobacter	0.0018	0.0000	0.2740	0.0000*	**0.0062**	0.0000*	0.0000*
Escherichia	0.0053	0.0000	0.0522	0.0000*	0.0000*	0.0000*	0.0000*
Fusobacterium	0.0246	0.0226	0.0264	**0.1893**	**0.4510**	**0.1944**	**0.1401**
Gemella	0.0188	0.0068	0.0036	0.0000*	0.0000*	0.0000*	0.0016*
Granulicatella	0.0166	0.0266	0.0123	0.0000*	0.0000*	**0.4896**	0.0000*
Haemophilus	0.0556	0.0703	0.0262	0.0000*	0.0004*	0.0000*	0.0000*
Klebsiella	0.0117	0.0000	0.0267	0.0000*	0.0000*	0.0000*	0.0000*
Leptotrichia	0.0057	0.0099	0.0162	0.0000*	**0.0020**	0.0000*	0.0016*
Megasphaera	0.0039	0.0096	0.0007	0.0000*	0.0000*	0.0000*	0.0000*
Neisseria	0.0325	0.0529	0.0551	0.0000*	0.0000*	0.0000*	**0.5568**
Oribacterium	0.0068	0.0096	0.0016	0.0000*	**0.0541**	0.0000*	0.0000*
Porphyromonas	0.0106	0.0143	0.0334	0.0000*	**0.0366**	0.0000*	0.0000*
Prevotella	0.2708	0.2506	0.1041	0.0000*	**0.0211**	0.0000*	0.0000*
Providencia	0.0000	0.0000	0.0161	0.0000*	**1.0000**	0.0000*	**1.0000**
Rothia	0.1116	0.0654	0.0310	0.0000*	0.0000*	0.0000*	0.0000*
Salmonella	0.0000	0.0000	0.0054	0.0000*	**1.0000**	0.0000*	**1.0000**
Selenomonas	0.0025	0.0087	0.0031	0.0000*	0.0000*	**0.1564**	0.0000*
Serratia	0.0000	0.0000	0.0519	0.0000*	**1.0000**	0.0000*	**1.0000**
Streptococcus	0.2754	0.2268	0.1409	0.0000*	0.0000*	0.0000*	0.0000*
TM7_genera_incertae_sedis	0.0048	0.0118	0.0033	0.0000*	0.0000*	**0.0075**	0.0000*
Veillonella	0.0780	0.1175	0.0279	0.0000*	0.0000*	0.0000*	0.0000*
