# Supplementary material for: Comparative analysis of the human saliva microbiome from different climate zones: Alaska, Germany, and Africa
Source: BMC Microbiol. 2014 Dec 17;14:316. doi: 10.1186/s12866-014-0316-1 (PMC4272767; doi:10.1186/s12866-014-0316-1)
Supplement: Additional file 6: Table S2. — Number of connections from the partial correlation network assigned to different phyla. [file 12866_2014_316_MOESM6_ESM.pdf]

Table S2 Number of connections

No. of positive interactions

Phylum	Alaska	Germany	Africa
Proteobacteria	24	15	16
TM7	3	5	3
Firmicutes	23	19	19
Actinobacteria	7	1	1
Fusobacterium	4	8	7
Bacteroidetes	11	10	14
Total	72	58	60

No. of negative interactions

Phylum	Alaska	Germany	Africa
Proteobacteria	30	19	14
TM7	2	2	0
Firmicutes	37	30	5
Actinobacteria	9	8	2
Fusobacterium	7	5	1
Bacteroidetes	11	8	2
Total	96	72	24

(Positive - Negative) interactions

Phylum	Alaska	Germany	Africa
Proteobacteria	-6	-4	2
TM7	1	3	3
Firmicutes	-14	-11	14
Actinobacteria	-2	-7	-1
Fusobacterium	-3	3	6
Bacteroidetes	0	2	12
Total	-24	-14	36
