# Supplementary material for: Comparative analysis of the human saliva microbiome from different climate zones: Alaska, Germany, and Africa
Source: BMC Microbiol. 2014 Dec 17;14:316. doi: 10.1186/s12866-014-0316-1 (PMC4272767; doi:10.1186/s12866-014-0316-1)
Supplement: Additional file 9: Table S3. — Comparison of 56 core OTUs with published results from HMP. [file 12866_2014_316_MOESM9_ESM.pdf]

Table S3 Comparison of the 56 common OTUs with the core OTUs described by Li et al, 2013 and Huse et al, 2012

(A) The taxonomy assignments and comparisons

OUT-ID	Assigned-phylum	Assigned-genus	Presented in Li et al, 2013	Presented in Huse et al, 2012
OTU-66	Actinobacteria	Actinomyces	Yes	Yes
OTU-1261		Rothia	No	Yes
OTU-68				
OTU-73				
OTU-289				
OTU-518				
OTU-7				
OTU-362				
OTU-203	Bacteroidetes	Porphyromonas	Yes	Yes
OTU-1162		Prevotella	Yes	Yes
OTU-2084				
OTU-189				
OTU-27				
OTU-219				
OTU-23				
OTU-240				
OTU-245				
OTU-292				
OTU-303				
OTU-384				
OTU-385				
OTU-508				
OTU-577				
OTU-59				
OTU-363	Firmicutes	Lactobacillus	No	Yes
OTU-2009				
OTU-1169		Streptococcus	Yes	Yes
OTU-1244				
OTU-1772				
OTU-265				
OTU-463				
OTU-5629				
OTU-1166				
OTU-117				
OTU-1258				
OTU-2147				
OTU-30				
OTU-344				
OTU-496				
OTU-5				
OTU-520				
OTU-276				
OTU-1469				
OTU-1114				
OTU-1267				
OTU-466				
OTU-611				
OTU-60		Veillonella	No	Yes
OTU-247				
OTU-504				
OTU-635				
OTU-672	Fusobacteria	Fusobacterium	Yes	Yes
OTU-2996		Leptotrichia	No	Yes
OTU-200	Proteobacteria	Haemophilus	No	No
OTU-223				
OTU-201		Neisseria	Yes	No

(B) The summary of taxonomy assignments and comparisons

Genus	Number	Presented in Li et al, 2013	Presented in Huse et al, 2012
Actinomyces	1	No	No
Fusobacterium	1	No	Yes
Lactobacillus	2	No	No
Haemophilus	2	No	No
Leptotrichia	1	No	Yes
Neisseria	1	No	No
Porphyromonas	1	No	No
Prevotella	15	No	No
Rothia	7	No	No
Streptococcus	21	Yes	Yes
Veillonella	4	No	Yes

Phylum	Number	Presented in Li et al, 2013	Presented in Huse et al, 2012
Actinobacteria	8	No	No
Bacteroidetes	16	Yes	Yes
Firmicutes	6	Yes	Yes
Fusobacteria	2	No	Yes
Proteobacteria	24	No	No
