# Supplementary material for: Comparative analysis of the human saliva microbiome from different climate zones: Alaska, Germany, and Africa
Source: BMC Microbiol. 2014 Dec 17;14:316. doi: 10.1186/s12866-014-0316-1 (PMC4272767; doi:10.1186/s12866-014-0316-1)
Supplement: Additional file 10: Table S4. — Results from subsampling. [file 12866_2014_316_MOESM10_ESM.pdf]

Table S4 Statistics results from subsampling.

(A) Subsampled ~2500 reads from ~12 individuals from each population

Group	Original		Sub-sampled						
	Number of Individuals	Number of Sequences	Number of Individuals	Number of Sequences	Number of OTUs	Number of Genera	Unknown (%)	Between individuals (%)	Within individuals (%)
Atqasuk	14	2661	14	2650	705	45	4.6	13.46	86.54
Barrow	40	10937	12	3333	849	44	3.2	8.88	91.12
Nuiqsut	13	2972	13	2608	719	41	3.7	8.23	91.77
Wainwright	9	2905	9	2344	714	47	4.1	6.71	93.29
Germans	10	4388	10	2479	574	51	2.8	2.34	97.66
BP	38	22948	12	3313	875	61	11.6	11.0	89.0
DRC	15	4503	15	3183	573	41	8.0	42.32	57.68
SL	13	16602	13	3593	768	43	10.7	31.48	68.52

(B) Subsampled 10 individuals from each group

Group		Number of Individuals	Number of Sequences	Number of OTUs	Number of Genera	Unknown (%)	Among groups (%)	Between individuals (%)	Within individuals (%)
Alaskans	Original	76	19475	2886	73	3.82	6.11	18.46	75.43
Germans		10	4388	887	58	3.05			
Africans		66	44053	4145	108	10.95			
Alaskans	Sub-sampled	10	2437	576	50	2.22	2.5	10.24	87.26
Germans		10	2479	464	52	0.78			
Africans		10	2496	537	53	2.32			
