# Supplementary material for: Comparative analysis of the human saliva microbiome from different climate zones: Alaska, Germany, and Africa
Source: BMC Microbiol. 2014 Dec 17;14:316. doi: 10.1186/s12866-014-0316-1 (PMC4272767; doi:10.1186/s12866-014-0316-1)
Supplement: Additional file 13: Figure S9. — Comparison of the alpha- and beta-diversity analysis from original and subsampled reads. [file 12866_2014_316_MOESM13_ESM.pdf]

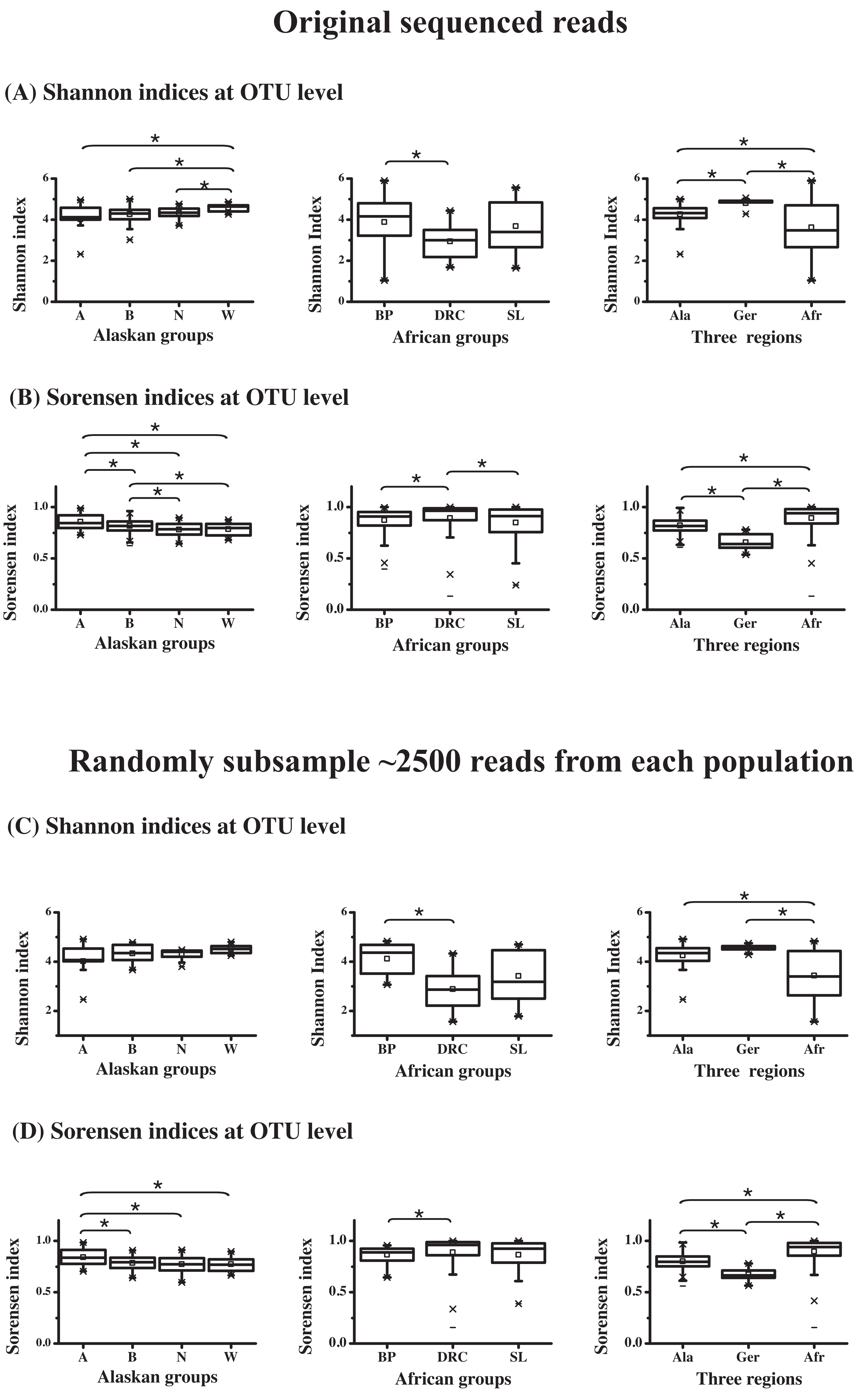
Original sequenced reads
(A) Shannon indices at OTU level
Shannon index
Alaskan groups
A
B
N
W
Shannon Index
African groups
BP
DRC
SL
Shannon Index
Three regions
Ala
Ger
Afr
(B) Sorensen indices at OTU level
Sorensen index
Alaskan groups
African groups
Three regions
Randomly subsample ~2500 reads from each population
(C) Shannon indices at OTU level
Shannon index
Alaskan groups
Shannon Index
African groups
Three regions
(D) Sorensen indices at OTU level
Sorensen index
Alaskan groups
African groups
Three regions
