# Supplementary material for: Comparative analysis of the human saliva microbiome from different climate zones: Alaska, Germany, and Africa
Source: BMC Microbiol. 2014 Dec 17;14:316. doi: 10.1186/s12866-014-0316-1 (PMC4272767; doi:10.1186/s12866-014-0316-1)

Randomly subsample 10 individuals with ~2500 reads from each group

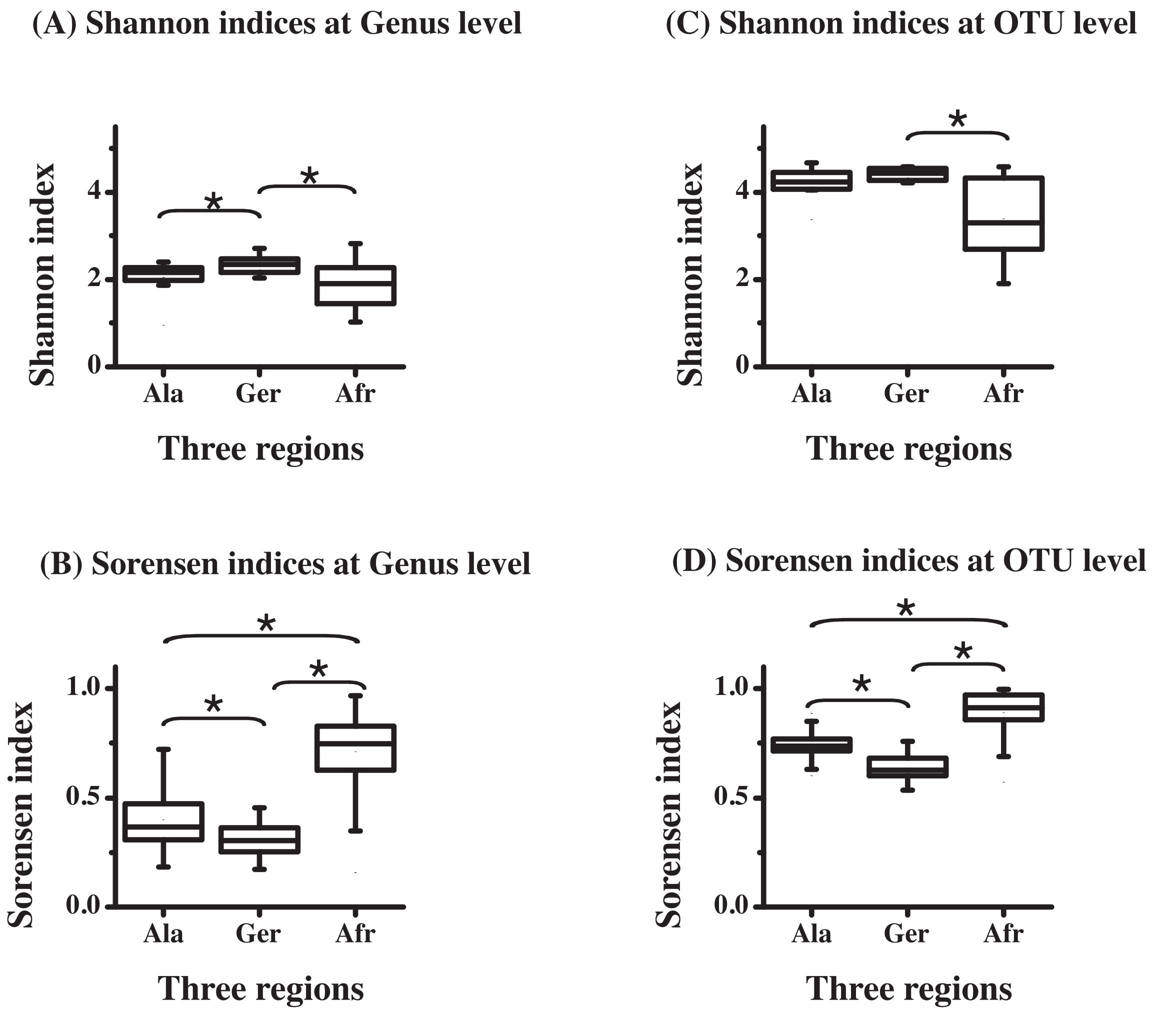

Supplement: Additional file 15: Figure S11. — Diversity analysis by subsampling 10 individuals from each group. [file 12866_2014_316_MOESM15_ESM.pdf]
